# Cryoablation-induced neutrophil Ca^2+^ elevation and NET formation exacerbate immune escape in colorectal cancer liver metastasis

**DOI:** 10.1186/s13046-024-03244-z

**Published:** 2024-12-09

**Authors:** Hongtong Tan, Yiquan Jiang, Lujun Shen, Gulijiayina Nuerhashi, Chunyong Wen, Ling Gu, Yujia Wang, Han Qi, Fei Cao, Tao Huang, Ying Liu, Weining Xie, Wuguo Deng, Weijun Fan

**Affiliations:** 1 Sun Yat-Sen University Cancer Center, State Key Laboratory of Oncology in South China, Guangdong Provincial Clinical Research Center for Cancer, Collaborative Innovation Center for Cancer Medicine, Guangzhou, China; 2 Guangdong Provincial Hospital of Integrated Traditional Chinese and Western Medicine, Guangdong, China

**Keywords:** Cryoablation, Neutrophils, Liver metastasis, Immunotherapy

## Abstract

**Background:**

Liver metastasis poses a significant barrier to effective immunotherapy in patients with colorectal cancer. Cryoablation has emerged as a vital supplementary therapeutic approach for these patients. However, its impact on the tumor microenvironment following the ablation of liver metastases remains unclear.

**Methods:**

We acquired multi-omics time-series data at 1 day, 5 days, and 14 days post-cryoablation, based on tumor and peripheral blood samples from clinical patients, cell co-culture models, and a liver metastases mouse model built on the MC38 cell line in C57BL/6 J mice. This dataset included single-cell transcriptomic sequencing, bulk tissue transcriptomic sequencing, 4D-Label-Free proteomics, flow cytometry data, western blot data, and histological immunofluorescence staining of pathological specimens.

**Results:**

We found that a neutrophil-related inflammatory state persisted for at least 14 days post-cryoablation. During this period, neutrophils underwent phenotypic changes, shifting from the N1 to the N2 type. Cryoablation also caused a significant increase in intracellular Ca^2+^ concentration in neutrophils, which triggered the formation of PAD4-dependent neutrophil extracellular traps (NETs), further promoting immune evasion. Moreover, animal studies demonstrated that depleting or inhibiting the CXCL2-CXCR2 signaling axis within neutrophils, or degrading NETs, could effectively restore the host’s anti-tumor immune response.

**Conclusions:**

These findings underscore the critical role of neutrophils and their NETs in immune escape following cryoablation. Targeting the CXCL2-CXCR2-Ca^2+^-PAD4 axis could enhance the therapeutic response to PD-1 antibodies, providing a potential strategy to improve treatment outcomes for colorectal cancer with liver metastases.

**Graphical Abstract:**

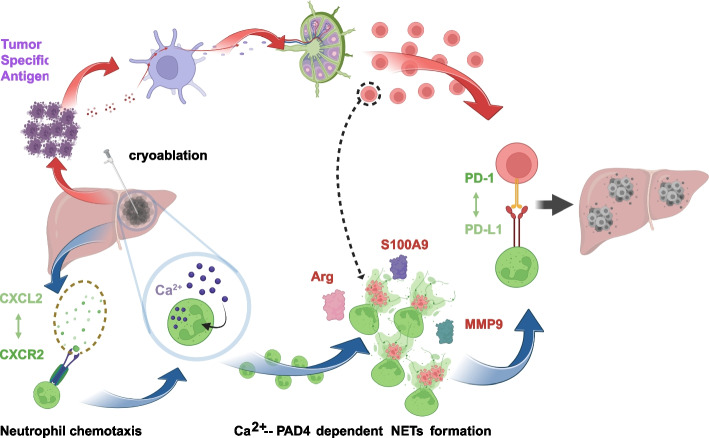

**Supplementary Information:**

The online version contains supplementary material available at 10.1186/s13046-024-03244-z.

## Introduction

Colorectal cancer is the third most common malignancy worldwide, with the liver being the primary metastasis site [1]. Evidence suggests that liver metastases promote resistance to immune checkpoint inhibitors [2]. A 2021 phase Ib/II trial demonstrated that colorectal cancer patients with liver metastases treated with regorafenib and tiragolumab had a significantly lower objective response rate compared to those without liver involvement (8.7% versus 30.0%) [3]. Existing evidence indicates that the immune microenvironment of colorectal cancer liver metastases is characterized by significant neutrophil infiltration, with these cells exhibiting both pro-tumor and anti-tumor properties [4]. Studies have highlighted that the functional heterogeneity of neutrophil subpopulations is a crucial factor influencing the efficacy of immunotherapy in patients with colorectal cancer liver metastases [5]. Historically, neutrophils were considered a short-lived, homogeneous, and unidimensional population with minimal significance in tumor progression and treatment response. However, with the widespread application of advanced multi-omics technologies, neutrophils have regained significant research interest. Recent single-cell transcriptomic data have demonstrated the heterogeneity of neutrophils in terms of gene expression, transcription factors, genetic characteristics, and developmental pathways [6]. Neutrophils are broadly divided into N1 and N2 phenotypes based on their differential effects on immunotherapy responses. Studies have shown that the N1 neutrophil phenotype is associated with high expression of Fas, TNF-α, and ICAM-1, typically accompanied by elevated infiltration levels of CD8^+^ T cells and NK cells, thereby enhancing anti-tumor immune responses. In contrast, N2 neutrophils are characterized by high expression of CXCR4, Arg-1, VEGF, and MMP-9, with their high levels of infiltration promoting tumor progression [7]. Research has indicated that the high infiltration levels of N2 neutrophils are a critical factor contributing to the dysfunction of NK cells or CD8^+^ T cells, ultimately leading to a reduced response rate to immunotherapy [8]. These findings suggest that neutrophils are crucial modulators of immunotherapy efficacy.

Cryoablation is an emerging therapeutic option that offers direct tumor cytotoxicity while preserving tumor antigens, thereby stimulating an anti-tumor immune response. This technique destroys tumor tissue through freeze–thaw cycles, leading to cell death. A meta-analysis of 15 studies revealed that cryoablation provides impressive local tumor control for liver metastases from colorectal cancer [9]. Preclinical models have demonstrated that cryoablation yields a synergistic effect when combined with immunotherapies, such as TLR agonists, PD-1, and CTLA-4 antibodies [10]. Our previous research observed that the combination of cryoablation with anti-PD1 therapy improved prognosis for some patients with liver metastases, increasing the overall response rate from 14.7% to 26.7% [11]. However, many patients still do not benefit from cryoablation, and the underlying reasons remain elusive.

It is well-known that cryoablation readily induces a strong localized inflammatory response. As early as 2009, a study reported significant infiltration of neutrophils both within and surrounding the ablation area following cryoablation [12]. However, the precise interactions between cryoablation and neutrophil function in the tumor microenvironment (TME) remain poorly understood. In recent years, the discovery of NETs has renewed interest in the role of neutrophils. Several critical questions remain unanswered, including whether and how the functional phenotype of neutrophils changes after cryoablation, whether cryoablation induces the formation of NETs, and whether NETs, as the final form of neutrophils, further weaken anti-tumor immunity.

To address these questions, we propose the following hypothesis: Cryoablation for colorectal cancer liver metastases not only directly induces tumor cell death but also presents tumor antigens, thereby activating the host’s anti-tumor immune response and enhancing the efficacy of immunotherapeutic agents. However, cryoablation also triggers a myeloid transformation associated with an inflammatory response, leading to the infiltration of a large number of neutrophils, which subsequently impacts anti-tumor immunity. To analyze dynamic changes in inflammatory and anti-tumor immune responses following cryoablation, we collected time-series data at multiple postoperative time points. This data included single-cell transcriptomic sequencing, pathological specimens and proteomic analysis. Through comprehensive analysis of these time-series data, we confirmed the temporal and spatial synchronization of neutrophil infiltration and NETs formation following cryoablation. We clarified the regulatory role of neutrophils and NETs in modulating anti-tumor immunity after cryoablation. In addition, we identified neutrophils and their NETs as targets to improve the efficacy of PD-1 monoclonal antibody treatment following cryoablation of colorectal cancer liver metastasis.

## Materials and methods

### Patients before and after cryoablation

We retrospectively collected routine blood test data from 49 patients with malignant liver tumors who underwent cryoablation at Sun Yat-sen University Cancer Center. Blood samples were obtained before and 7 days after cryoablation. The Wilcoxon signed-rank test was used for the comparison of NLR, while the absolute neutrophil counts were analyzed using the paired T-test.

### Single-cell RNA-seq analysis

Quality control of the expression matrices for each sample was performed using Seurat v4.3.0 software. The DoubletFinder v2.0.2 package was used to remove doublets. After quality control, RNA data was scaled using the ScaleData function in Seurat. Principal component analysis was conducted on the top 2,000 highly variable genes to reduce dimensionality. The first 50 principal components were used for batch correction with the Harmony v1.0 algorithm and subsequently in the FindNeighbors and FindClusters functions (resolution = 1.0) in Seurat to identify cell clusters. The identified clusters were visualized and analyzed using UMAP. Cluster-enriched genes were identified using the FindAllMarkers function in Seurat with a minimum percentage threshold of 0.25. Gene function enrichment analysis was conducted using the R package clusterProfiler v3.14.0, with parameters pvalueCutoff, qvalueCutoff, and pAdjustMethod set to 1, 1, and “BH”, respectively.

### Single-cell pseudotime analysis

We used Monocle 2 v2.28.0 for pseudotime analysis. The pseudotime starting point was determined using CytoTRACE v0.3.3. Genes with high dispersion across cells were used to select and order the cells of interest. The data were partitioned into two components using the DDRTree approach, and branch-dependent genes were identified using BEAM.

### Cell–cell interaction analysis

We used CellChat v2.1.0 to infer and visualize intercellular communication based on the expression of known ligand-receptor pairs. The netVisual_bubble function in CellChat was used to visualize differences in communication probabilities for ligand-receptor pairs, considering both interaction directions.

### Gene Set Variation Analysis (GSVA)

To investigate the clinical significance of neutrophil subpopulations associated with NETs, we performed gene set variation analysis on the scRNA-seq data. The GSVA v1.46.0 R package was used for this analysis. Details and references for the GSVA analysis are provided in Supplementary Table S1.

### Bulk-RNA-seq analysis

Total RNA was extracted using TRIzol reagent (Thermo Fisher Scientific). For library construction, 1 μg of RNA was prepared using the VAHTS® Universal V8 RNA-seq Kit (Vazyme Technology) and sequenced on a Novaseq 6000 instrument in a 2 × 150 PE configuration. Sequencing reads were processed with fastp to eliminate poor quality reads, adapter contamination and high N-content.

Mapped reads were aligned using HISAT and Bowtie2, and gene expression was analyzed with RSEM. Expression data underwent correlation, clustering, and PCA analyses using core R functions and ggplot2. Differentially expressed genes were identified using DEGseq with specified parameters based on the Poisson distribution.

### Proteomic detection

Samples were lysed and proteins were extracted using SDT buffer (4% SDS, 100 mM Tris–HCl, 1 mM DTT, pH 7.6). Protein concentration was measured using a BCA protein assay kit (Bio-Rad). Proteins were digested with trypsin using the FASP method. The digested peptides were desalted in a C18 column (Empore™ SPE Cartridges C18 (standard density), bed ID 7 mm, volume 3 ml, Sigma), vacuum-centrifuged, and reconstituted in 40 μl of 0.1% (v/v) formic acid.

Each sample (20 μg of protein) was mixed with 5 × loading buffer, boiled for 5 min, and separated by 12.5% SDS-PAGE (constant current 14 mA, 90 min) [13]. Proteins were visualized using Coomassie Blue R-250 staining.

For LC–MS/MS analysis, a Q Exactive mass spectrometer (Thermo Scientific) was used for 60/120/240 min, coupled with an Easy nLC system (Thermo Fisher Scientific). Raw MS data from each sample were normalized and combined. Identification and quantitative analysis were conducted using MaxQuant 1.5.3.17 software.

### Quantitative detection of cytokines

We detected 32 cytokines, including G-CSF, GM-CSF, M-CSF, CXCL1, CXCL2, CXCL5, CXCL9, CXCL10, CCL2, CCL3, CCL4, CCL5, CCL11, IL-1α, IL-1β, IL-2, IL-3, IL-4, IL-5, IL-6, IL-7, IL-9, IL-10, IL-12p40, IL-12p70, IL-13, IL-15, IL-17, TNF-α, LIF, IFN-γ, and VEGF, using Luminex xMAP® liquid suspension array technology; for detailed methods, see Supplementary Figure S1.

### Cell culture

The human promyelocytic leukemia cell line HL-60 was obtained from the American Type Culture Collection (ATCC). The colorectal cancer cell line MC38 was kindly provided by Professor Musheng Zeng of the Sun Yat-sen University Cancer Center.

Both cell lines were cultured in their respective media: RPMI 1640 for HL-60 and DMEM for MC38. Each medium was supplemented with 10% fetal bovine serum (Invitrogen) and 1% penicillin/streptomycin (Gibco). Cells were incubated at 37 °C in a humidified incubator with 5% CO_2_. For HL-60 cells, 1.3% DMSO was added to the complete medium for 3 consecutive days to induce differentiation into a neutrophil-like phenotype, referred to as differentiated HL-60 cells.

### Animal models

In this study, the in vivo experiments were divided into three parts. The first part focused on a time-series analysis after cryoablation treatment of colorectal cancer liver metastasis. The second part examined the effects of neutrophil or NETs antagonism on antitumor immunity following cryoablation, while the third part explored the impact of neutrophils on the abscopal effect post-cryoablation.

For all in vivo experiments, wild-type male C57BL/6 J mice (SPF grade, 5–7 weeks old, B6, Gempharmatech) were used. Tumor size was monitored, and mice were randomly grouped when tumors reached 5–7 mm in diameter, which typically occurred about 10 days after tumor cell inoculation. MC38/wild-type cells in the logarithmic growth phase were harvested after 3–4 passages and injected into the subcapsular region of the mouse liver (5.0 × 10^5^ cells per mouse) to establish the colorectal cancer liver metastasis model. Additionally, the subcutaneous tumor model was created by injecting 1.0 × 10^6^ cells bilaterally into the shoulder-back regions of mice.

For the first part of the experiment, mice with colorectal cancer liver metastasis underwent partial cryoablation, and samples were collected at three specific time points: on Day 1, Day 5, and Day 14 post-surgery. In the second and third parts, tumor implantation, cryoablation, drug administration, and sample collection were carried out at pre-defined time points. Detailed information regarding the timing of tumor implantation, cryoablation, administration of specific inhibitors, and sample collection is provided in Supplementary Figure S6 and its legend. All animal studies were approved by the Animal Care and Use Committee of Sun Yat-sen University Cancer Center.

### Cryoablation and administration of drugs in mice

Cryoablation was performed using the Visual-ICE™ System (Galil Medical, Israel). In the subcutaneous tumor-bearing mouse model, the cryoprobe was inserted into the targeted lesions, and two 20-s cycles, separated by a 10-s freezing and an active 10-s thawing session, were performed. The procedure was repeated until complete tumor ablation was achieved. In the colorectal cancer liver metastasis mouse model, the cryoprobe was inserted into the targeted lesions, and a single 10-s cycle was performed, with a 5-s freezing period and a 5-s active thawing session in between. Incomplete tumor ablation was achieved.

For in vivo phenotypic experiments in mice, five drugs were used: anti-PD1 (10 mg/kg, Clone: RMPI1-14, BioxCell), anti-Ly6G (15 mg/kg, Clone: 1A8, BioLegend), DNase I (15 mg/Kg, Roche), GSK484 HCl (25 mg/kg, Selleck), and anti-CXCR2 (15 mg/kg, Selleck). Anti-Ly6G has been reported to effectively deplete neutrophils in C57BL/6 J mice, with a sustained effect lasting up to approximately 4 weeks [14]. Therefore, we selected anti-Ly6G as the primary drug to deplete intratumoral neutrophil infiltration. However, other studies have observed that in some mice with impaired complement/macrophage pathways, the depletion effect of anti-Ly6G on neutrophils showed slight variations [15]. To minimize the impact of this issue on the experiment, we further included anti-CXCR2, a well-validated drug that blocks neutrophil infiltration into tumors. Anti-CXCR2 functions primarily by blocking the CXCR2 receptor, thus preventing the migration of bone marrow-derived neutrophils into the tumor [16].

DNase I has been shown to directly degrade NETs and promote anti-tumor immunity [17, 18]. Furthermore, Ca^2+^-dependent PAD4 mediates NETs formation, which has been identified as the main mechanism for NETs generation. Inhibiting PAD4 has been validated as an effective strategy to block NETs formation [19, 20]. Therefore, we selected DNase I and anti-PAD4 as two drugs to inhibit the intratumoral infiltration of NETs following cryoablation. All five drugs were administered by intraperitoneal injection. For anti-CXCR2 administration, the drug was prepared by dissolving the powdered substance sequentially in 2% DMSO, 30% PEG300, 5% Tween-80, and ddH_2_O.

### Preparation of single-cell suspensions

Tumor tissues were cut into approximately 2 × 2 mm pieces. A single-cell suspension was prepared using the Mouse Tumor Dissociation Kit (Miltenyi) following the manufacturer’s instructions.

### Detection of proteins in CD8^+^ T cells and NK cells

Cells were treated with a Leukocyte Activation Cocktail (2 μl/ml, BD) and incubated for 4 h. After lysing the erythrocytes, a viability dye was added and incubated. The suspension was washed, treated with anti-mouse CD16/CD32 antibody (Clone: 2.4G2, BD), and incubated on ice for 5 min. The cells were then stained with anti-mouse CD45 (FITC, BD), CD8 (APC-A700, BioLegend), CD4 (BV510, BD), NK1.1 (BV650, BD), and PD-1 (PC5.5, BD), and incubated for 30 min.

After washing and fixing, cells were permeabilized three times using Intracellular Staining Perm Wash Buffer. They were stained with anti-mouse IFN-γ (PE-Cy7, BD), TNF-α (BV421, BD), and Perforin (APC, BioLegend). Staining was conducted in the dark for 30 min.

### Detection of Neutrophils and NETs

Neutrophil detection: After lysing erythrocytes, cells were resuspended in 100 μl of viability dye to distinguish live cells. After washing and blocking, cells were stained with anti-mouse CD45 (FITC, BD), CD11b (BV605, BD), Ly6G (PE, BioLegend), and Ly6C (BV421, BioLegend) for 30 min in the dark.

NET detection: Erythrocytes were lysed and centrifuged at 100 g for 10 min. Cells were resuspended in 2% paraformaldehyde and incubated for 20 min at room temperature. After centrifugation at 100 g for 10 min, cells were resuspended in 100 μl PBS and stained with anti-mouse CD45 antibody (APC-Cy7, BD), Ly6G (APC, BD), DAPI (BD), and SytoxOrange dead cell stain (Invitrogen). After a 20-min incubation in the dark at room temperature, 300 μl of PBS was added for final detection. The gating strategy was performed using FlowJo software (v10.1, FlowJo).

### Immunofluorescence staining

Three-micrometer thick sections were deparaffinized and treated with heat-induced antigen retrieval using Tris–EDTA (pH9.0) solution. After blocking, primary antibodies were added and incubated overnight at 4 °C. Subsequently, second antibodies and different fluorescent markers were applied according to the protocol (Panovue). DAPI (Thermo Fisher) or Hoechst 33,342 (Thermo Fisher) was then added. Antibodies were removed by washing in PBS containing 0.5% Tween-20 (Millipore). Finally, slides were mounted with ProLong Gold anti-fade reagent (Thermo Fisher) and cover-slipped. Slides were scanned at 40 × magnification using the Vectra Polaris automated digital slide scanner.

The primary antibodies used included anti-mouse Ly6G (ab238132, Abcam), CD11b (ab133357, Abcam), CD8α (ab217344, Abcam), neutrophil elastase (90120S, Cell Signaling Technology), H2B (ab52599, abcam), and histone H3 (4499 T, Cell Signaling Technology). We used fluorescent dyes with emission wavelengths of 520, 570, and 690 nm in our experiments. Scanned results were analyzed using Halo analysis software (Indica Labs) and ImageJ software.

### In vitro simulation of cryoablation

After centrifugation to obtain the cell pellet, we adjusted the cell concentration and re-suspended the pellet in 1 ml of complete medium. The suspension was transferred to a 1.5 ml EP tube, then placed into a 15 ml centrifuge tube containing 13.5 ml of isopropanol. The tubes were quickly transferred into liquid nitrogen to simulate rapid freezing for a few minutes and then moved swiftly to a thermostatic bath to simulate rapid thawing. The Supplementary Figure S2A represents a schematic diagram of the process. Detailed times and temperatures for freezing and thawing steps are provided in the figure legend.

### qPCR Analysis of Cxcl2

After in vitro cryoablation, the MC38 cells were replated. After 3 days of cultivation, the cells were collected using a cell scraper. Total cellular RNA was extracted (ES Science) and reverse transcribed (ES Science) to obtain cDNA. The transcriptional level of Cxcl2 was detected using the Roche LightCycler® 480 fluorescence quantitation system. Gene expression was normalized to Gapdh. Mouse Gapdh primer sequences: Gapdh-F: AGGTCGGTGTGAACGGATTTG; Gapdh-R: TAGACCATGTAGTTGAGGTCA. Mouse Cxcl2 primer sequences: Cxcl2-F: CATAGCCACTCTCAAG; Cxcl2-R: CCTTTGTTCAGTATCTT.

### Western blot assay

Cells were collected in pre-cooled PBS, centrifuged, and lysed on ice for 30 min in a lysis buffer containing protease inhibitors. This process yielded protein extracts. Protein concentration was measured using the BCA Protein Assay Kit (Bio-Rad). Equal amounts of protein were loaded onto an SDS-PAGE gel and transferred to a PVDF membrane. The membrane was blocked with PBS containing 0.5% Tween-20 and 5% non-fat milk, followed by overnight incubation at 4 °C with specific primary antibodies. After washing the membrane the next day, it was incubated with secondary antibodies for 1 h at room temperature. Finally, the membrane was washed again, and a Thermo Fisher Scientific chemiluminescent kit was used to detect immune complexes.

The primary antibodies used were: anti-human neutrophil elastase (1:3000, Abcam), Histone H3 (1 μg/ml, Abcam), PAD4 (1:2000, Abcam), and anti-mouse Cxcl2 (2.5 μg/ml, Thermo Fisher Scientific).

### Determination of Intracellular Ca^2+^ Concentration in Neutrophils

Neutrophils from human peripheral blood were sorted and resuspended in HHBS buffer. Cells from both the control group and the cryoablation group were evenly distributed onto 6 cm^2^ culture dishes. Under light-protected conditions, Pluronic® F-127 (5 μl/ml, Invitrogen) and Fluo 4-AM (5 μM, Dojindo) were added to the culture system and incubated for 1 h. After three washes with HHBS buffer, the cells were resuspended in fresh HHBS and incubated for an additional 30 min. The cells were then observed using a fluorescence microscope and further analyzed by flow cytometry. The excitation wavelength was set to 494 nm and the emission wavelength to 516 nm.

### Statistical analysis

Sample sizes were indicated in the figure legends, and data were typically presented as the mean ± standard deviation. Statistical significance was assessed using either a two-tailed Mann–Whitney U test or Student’s t-test, as appropriate. Tumor growth curves were compared using one-way analysis of variance (ANOVA). P values less than 0.05 were considered statistically significant. GraphPad Prism 10.0.0 was used for statistical analysis. All experiments were repeated at least twice to ensure the reliability.

## Results

### Cryoablation Induces the Increase and Infiltration of N2-Type Neutrophils and NETs

Cryoablation is a valuable treatment for colorectal cancer liver metastasis, especially when combined with PD-1 monoclonal antibody therapy. However, many patients exhibit resistance, highlighting the need to identify targets that can enhance efficacy and improve treatment durability.

**Fig. 1:**
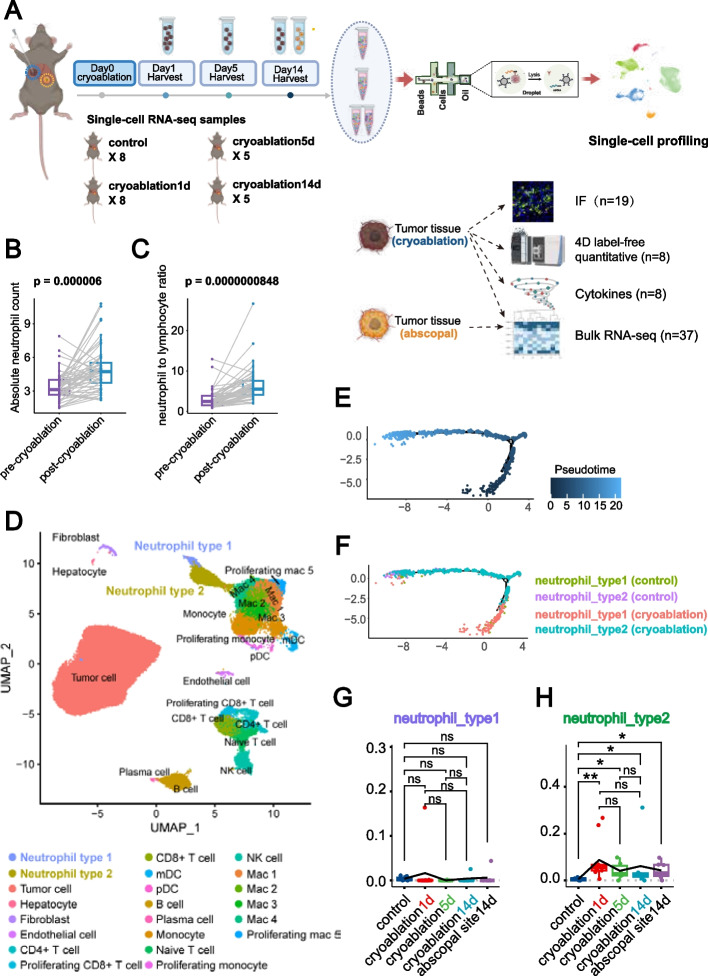
Cryoablation Induces Neutrophil Infiltration and Phenotypic Shift in Colorectal Cancer Liver Metastasis. **A** Overview of multi-omics data sources in this study. Thirty-one mice with MC38 cell line-colonized colorectal cancer liver metastasis were divided into four groups: control (*n* = 8), 1 day post-cryoablation (*n* = 10), 5 days post-cryoablation (*n* = 6), and 14 days post-cryoablation (*n* = 7). Each mouse had two tumors colonized on the largest liver lobe; one tumor was treated with incomplete cryoablation, while the other was left untreated. Mice were sacrificed at the specified time points for single-cell RNA sequencing, bulk RNA-seq, immunofluorescence staining, 4D-Label-Free proteomics, and cytokines quantification. For details of the cryoablation procedure, refer to the “Cryoablation and Administration of Drugs in Mice” section in Materials and Methods. Sample usage records are in Supplementary Table S2. **B** Retrospective analysis of blood test results from patients with colorectal cancer liver metastasis at Sun Yat-sen University Cancer Center shows a significant increase in absolute neutrophil count in peripheral blood post-cryoablation. **C** The neutrophil-to-lymphocyte ratio (NLR) significantly increases in the peripheral blood of patients after cryoablation compared to pre- treatment levels. **D** UMAP dimensionality reduction and clustering analysis of single-cell RNA sequencing data from cryoablation-treated and control samples identifies increased intratumoral neutrophil infiltration post-cryoablation. **E**,** F** Pseudotime analysis of single-cell RNA sequencing data from cryoablation and control groups reveal a developmental trajectory shift in neutrophils from N1-type to N2-type post-cryoablation. **G**,** H** Bulk RNA-seq analysis of samples from mice with colorectal cancer liver metastasis treated with cryoablation at 1, 5, and 14 days post-cryoablation shows no significant change in N1-type neutrophil infiltration, while N2-type neutrophil infiltration significantly increases

While cryoablation induces strong local inflammatory responses, research on the interplay between this inflammation and anti-tumor immunity has progressed slowly in recent years. Elevated neutrophil to lymphocyte ratio (NLR) is a significant biomarker associated with poor prognosis in colorectal cancer liver metastasis patients [21]. The role of neutrophils in regulating tumor immunity is not well understood, with conflicting reports. An increasing number of studies have shown that neutrophils exhibit remarkable plasticity within the tumor microenvironment. Although recent transcriptomic data suggest that neutrophils in the TME may possess more subtypes, a consensus has yet to be reached. The widely accepted view is that, based on a functional dichotomy, neutrophils can be roughly categorized into N1 and N2 types [22–24]. N1-type neutrophils are characterized by higher levels of TNF-α, granzyme B, and IFN-γ secretion, while expressing lower levels of IL-10 and Arg-1. Their primary function is to promote CD8^+^ T cell activation and enhance anti-tumor immune responses. In contrast, N2-type neutrophils express higher levels of immunosuppressive molecules such as Arg-1, IL-10, and TGF-β, thus inhibiting anti-tumor immunity. Additionally, N1-type neutrophils form fewer neutrophil extracellular traps (NETs), whereas N2-type neutrophils are actively involved in NETs formation [25]. Characterizing these subtypes in the TME of colorectal cancer liver metastasis following cryoablation could optimize immunotherapy strategies. Furthermore, we acquired multi-timepoint and multi-omics data. Details of the experimental workflow and data sources are provided in Fig. 1A.

We analyzed clinical data from 49 patients with colorectal cancer liver metastases treated with cryoablation at our hospital, revealing significant increases in neutrophil count and NLR in peripheral blood post-cryoablation (Fig. 1B, C). To gain deeper insights into neutrophil infiltration and phenotypic changes within the TME post-cryoablation, we established a mouse model and performed single-cell RNA sequencing and analysis.

Dimensionality reduction clustering and pseudotime analysis showed a significant increase in N2-type neutrophils within the TME in the cryoablation area (Fig. 1D). Pseudotime analysis shows that neutrophils develop into N2-type neutrophils after cryoablation (Fig. 1E, F). The number of N2-type neutrophils significantly increased on the first day post-cryoablation, persisting until day 14, while changes in N1-type neutrophils were minimal (Fig. 1G, H).

**Fig. 2:**
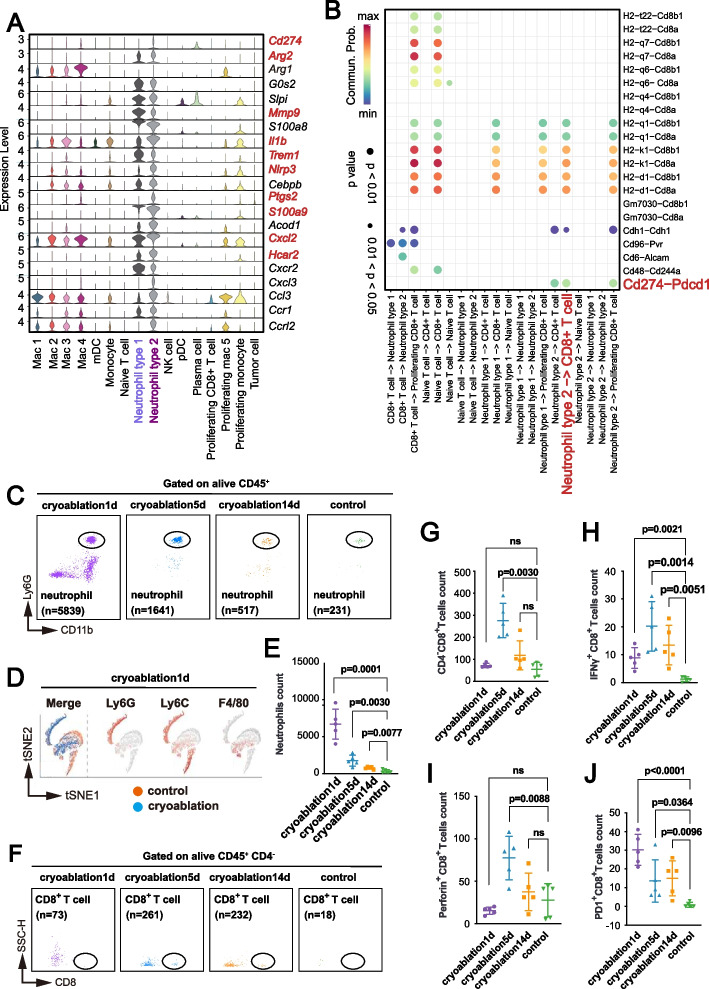
Cryoablation Reshapes CD8^+^ T Cell Anti-Tumor Immunity and Enhances CD274-CD279 Interaction Between N2-type Neutrophil and CD8^+^ T Cell. **A** Single-cell RNA sequencing shows that cryoablation upregulates immunosuppressive molecules such as IL-1β, S100A8, and S100A9 in N2-type neutrophils in colorectal cancer liver metastasis. **B** CellChat analysis of single-cell RNA sequencing data shows enhanced interaction between CD274 (PD-L1) on N2-type neutrophils and CD279 (PD-1) on CD8^+^ T cells post-cryoablation. **C**,** D** Flow cytometry shows significantly increased neutrophil infiltration in the TME of colorectal cancer liver metastasis post-cryoablation compared to controls, with the most pronounced increase on the first day. **E** Neutrophils are identified as live CD45^+^ CD11b^+^ Ly6G^+^ cells, showing elevated infiltration persisting for 14 days post-cryoablation. Each dot represents an independent biological sample (*n* = 5 mice per group), and data are shown as mean ± s.d. Significance was determined using p-values. **F**, **G** CD8^+^ T cell infiltration in the TME peaks 5 days post-cryoablation (*n* = 5 mice). Data are presented as mean ± s.d. Significance was determined using p-values, with “ns” indicating no significant difference. **H**,** I** Cryoablation restores CD8^+^ T cell tumor-killing ability in the TME, as evidenced by increased infiltration of IFNγ-expressing and perforin-expressing CD8^+^ T cells. Representative images of flow cytometry are in Supplementary Figure S2B (*n* = 5 mice). Data are mean ± s.d. Significance was determined using *p*-values, with “ns” indicating no significant difference. **J** Cryoablation increases PD-1 receptor expression on CD8.^+^ T cells in the TME of colorectal cancer liver metastasis. Each dot represents an independent biological sample (*n* = 5 mice per group), and data are shown as mean ± s.d. Significance was determined using p-values. Representative images of flow cytometry can be found in Supplementary Figure S2B. Group names indicate the day post-cryoablation sacrifice (1d, 5d, 14d) and the control group (sham-operated)

Further analysis revealed that N2-type neutrophils express higher levels of immunosuppressive proteins such as IL-1β, S100A8, S100A9, and Arg-2 (Fig. 2A). These genes are closely associated with NETs [26]. Receptor-ligand interaction analysis indicated enhanced interaction between CD279 and CD274 receptor-ligand pairs on neutrophils and CD8^+^ T cells following cryoablation (Fig. 2B).

### Cryoablation Restores CD8^+^ T Cell Cytotoxicity and Induces NETs Formation

To validate our time-series findings, we used a mouse model of colorectal cancer liver metastasis. Samples were collected at three post-cryoablation time points: day 1, day 5, and day 14. Flow cytometry revealed a significant and sustained increase in neutrophil infiltration within the TME at the cryoablation site, beginning on day 1 and persisting through day 14 (Fig. 2C-E). We also observed a significant increase in the infiltration of total CD8^+^ T cells, IFNγ^+^ CD8^+^ T cells, and perforin^+^ CD8^+^ T cells within the TME (Fig. 2F-I, Supplementary Figure S2B), suggesting that cryoablation restored the cytotoxic ability of CD8^+^ T cells against tumor cells. Interestingly, following cryoablation, CD8^+^T cells exhibited a significant upregulation in PD-1 receptor expression (Fig. 2J, Supplementary Figure S2B), highlighting the potential benefit of combining PD-1 monoclonal antibody therapy with cryoablation.

The in vitro co-culture model demonstrated that while cryoablation facilitated CD8^+^ T cell proliferation, neutrophils hindered this expansion and led to a notable increase in CD8^+^ T cell apoptosis (Supplementary Figure S3A-F). Cryoablation enhanced the cytotoxic capability of CD8^+^ T cells, evidenced by increased expression of IFNγ and TNFα (Supplementary Figure S4A-B), but neutrophils limited this potential (Supplementary Figure S4C-D).

**Fig. 3:**
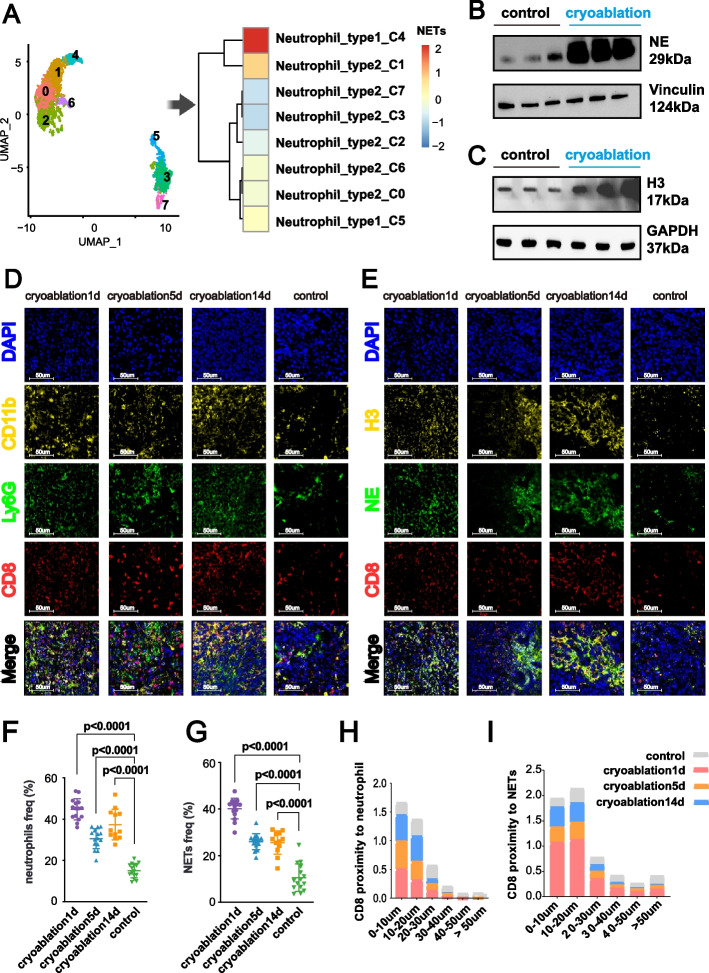
Cryoablation Promotes NETs Formation and Alters Spatial Dynamics of CD8 + T Cells in the TME. **A** Single-cell RNA sequencing of colorectal cancer liver metastasis tissues post-cryoablation reveals neutrophils tend towards a NETs state, indicated by GSVA scores. The specific gene set used is detailed in Supplementary Table S1. **B**,** C** Using differentiated HL-60 cells as human neutrophil substitutes, western blot analysis shows increased expression of NETs markers, neutrophil elastase (NE) and histone H3, after in vitro cryoablation (5 min freezing, 4 min thawing at 50 °C). A schematic of the in vitro cryoablation process is in Supplementary Figure S2A. **D** Immunofluorescence images of CD11b (yellow), Ly6G (green), and CD8 (red) in mouse colorectal cancer liver metastasis tissues at 1 day, 5 days, and 14 days post-cryoablation and control. DAPI (blue) indicates nuclei. CD11b and Ly6G co-expression define neutrophils, and CD8 positive expression defines CD8^+^ T cells. Scale bar = 50 μm. **E** Immunofluorescence images of NE (green), histone H3 (yellow), and CD8 (red) in mouse colorectal cancer liver metastasis tissues at 1 day, 5 days and 14 days post-cryoablation and control. DAPI (blue) indicates nuclei. NE and H3 co-expression define NETs, and CD8 positive expression defines CD8^+^ T cells. Scale bar = 50 μm. **F**,** G** Statistical analysis of neutrophil and NETs infiltration levels post-cryoablation. Three ROIs per sample, 15 ROIs for cryoablation1d, 15 ROIs for cryoablation5d, 12 ROIs for cryoablation14d, and 15 ROIs for control. Data analyzed using ImageJ, presented as mean ± s.d. Significance determined using p-values. **H**,** I** Proportion of CD8^+^ T cells within different distances from neutrophils and NETs, showing cryoablation’s impact on CD8^+^ T cell distribution. Spatial analysis using Halo software categorizes distances into 0–10 μm, 10–20 μm, 20–30 μm, 30–40 μm, 40–50 μm, and > 50 μm. Results shown as stacked bar charts: gray (control), red (cryoabaltion1d), yellow (cryoablation5d), and blue (cryoablation14d). Details in Supplementary Figure S4E

Recent studies have shown that NETs, the terminal form of neutrophils, can continuously weaken anti-tumor immunity after neutrophil death [27]. While no current evidence shows that cryoablation induces NETs formation, we investigated this possibility. We reclustered the cells in an unsupervised manner, resulting in eight distinct clusters. Gene Set Variation Analysis (GSVA) showed that Cluster 4, comprised of N1-type neutrophils, exhibited the highest score, suggesting that cryoablation may induce a phenotypic shift in these already scarce cells, pushing them from a protective state towards a more detrimental phenotype, closer to the NETs state (Fig. 3A). Although both N1 and N2-type neutrophils tend to evolve into NETs after cryoablation, N2-type neutrophils are the primary source of NETs due to their higher numbers compared to N1-type neutrophils after cryoablation.

We used differentiated HL-60 cells (dHL60) as a surrogate for neutrophils and simulated the cryoablation in vitro [28]. Western blot analysis indicated that cryoablation upregulated the expression of NETs markers [29, 30], neutrophil elastase (NE) and histone H3, in dHL60 cells (Fig. 3B, C).

### Spatial Colocalization between Neutrophils, NET and CD8^+^ T Cells within the TME Post-Cryoablation

In cell biology, distances below 100 µm between two cells or structures indicate potential for biological interactions [31]. However, the interactions between increased neutrophils/NETs and CD8^+^ T cells following cryoablation in colorectal cancer liver metastases are unknown. The dynamics of their spatial associations with CD8^+^ T cells within the TME across post-cryoablation inflammatory phases remain unclear.

We performed immunofluorescence staining on tumor samples from mice with colorectal cancer liver metastasis at different time points after cryoablation (Fig. 3D-G, Supplementary Figure S7A-B). Using Halo software, we conducted spatial analysis to assess the relationships between neutrophils/NETs and CD8^+^ T cells following cryoablation. The results showed significantly higher infiltration of neutrophils and NETs within the cryoablation lesion compared to the control group (Fig. 3D-G, Supplementary Figure S7A-B).

We used Halo software to conduct a spatial analysis of neutrophils/NETs and CD8^+^ T cells, calculating the proportion of CD8^+^ T cells surrounding neutrophils or NETs within various distance ranges (0–10 μm, 10–20 μm, 20–30 μm, 30–40 μm, 40–50 μm, and > 50 μm) as a percentage of total CD8^+^ T cells across all distances at each post-cryoablation time point. Most CD8^+^ T cells within cryoablated lesions were located within 50 μm of neutrophils/NETs (Fig. 3H, I, Supplementary Figure S4E).

Our results indicated that after cryoablation, CD8^+^ T cells in the TME are confined around neutrophils and NETs, limiting their cytotoxic effects on tumor cells, with this physical barrier lasting at least 14 days post-cryoablation. These findings indicate that neutrophil and NETs dynamics post-cryoablation are consistent across temporal and spatial dimensions, which may significantly influence long-term treatment outcomes in colorectal cancer liver metastasis patients.

### Reducing Neutrophil or NETs Infiltration Enhances Anti-Tumor Immunity After Cryoablation

Liver metastases create an “immune desert,” posing a persistent therapeutic challenge in immunotherapy [2]. To investigate whether neutrophils and NETs could enhance PD-1 antibody efficacy following cryoablation in colorectal cancer liver metastasis, we established four cohorts of mice bearing colorectal cancer liver metastases derived from the MC38 cell line. We employed two neutrophil-depleting agents (anti-Ly6G and SB225002, a CXCR2 receptor inhibitor), and two NETs-depleting agents (DNase I and GSK484 HCl, a protein arginine deiminase 4 (PAD4) inhibitor).

**Fig. 4:**
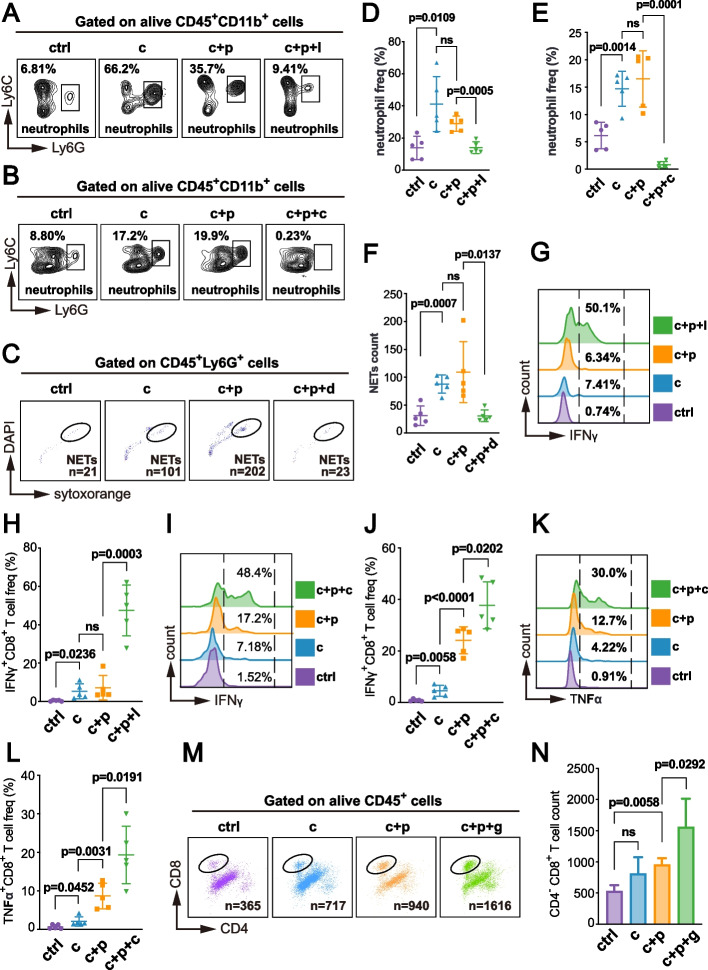
Inhibiting Neutrophil or NETs Infiltration Enhances Adaptive Anti-Tumor Immunity of CD8^+^ T Cells Post-Cryoablation. The experimental flowchart for the four mouse cohorts is in Supplementary Figure S6, and cryoablation parameters and drug administration protocols are detailed in the “Materials and Methods” section. Group abbreviations: “ctrl” control, “c” (cryoablation), “c + p” (cryoablation + anti-PD1), “c + p + l” (cryoablation + anti-PD1 + anti-Ly6G), “c + p + c” (cryoablation + anti-PD1 + anti-CXCR2), “c + p + d” (cryoablation + anti-PD1 + DNase I), “c + p + g” (cryoablation + anti-PD1 + GSK484 HCl). **A** Flow cytometry plots showing intratumoral infiltration levels of neutrophils (defined as live CD45^+^ CD11b^+^ Ly6G^+^ Ly6C^high^) in the ctrl, c, c + p, and c + p + l groups. **B** Flow cytometry plots showing intratumoral infiltration levels of neutrophils (defined as live CD45^+^ CD11b^+^ Ly6G^+^ Ly6C^high^) in the ctrl, c, c + p, and c + p + c groups. **C** Flow cytometry plots showing intratumoral infiltration levels of NETs (defined as CD45^+^ Ly6G^+^ DAPI^+^ SytoxOrange^+^) in the ctrl, c, c + p, and c + p + d groups. **D-F** Statistical analysis of neutrophils and NETs infiltration levels in the TME of the ctrl, c, c + p, c + p + l, c + p + c, and c + p + d groups. **G-J** Flow cytometry representative plots and statistical analysis of IFNγ-expressing CD8^+^ T cell infiltration levels in the TME of the ctrl, c, c + p, c + p + l, and c + p + c groups. **K**,** L** Flow cytometry representative plots and statistical analysis of TNFα-expressing CD8^+^ T cell infiltration levels in the TME of the ctrl, c, c + p, and c + p + c groups. **M**,** N** Flow cytometry representative plots and statistical analysis of CD8^+^ T cell infiltration levels in the TME of the ctrl, c, c + p, and c + p + g groups

These agents effectively reduced the intratumoral infiltration of neutrophils or NETs associated with cryoablation (Fig. 4A-F). Following cryoablation, anti-Ly6G significantly increased the infiltration of IFNγ^+^ CD8^+^ T cells (Fig. 4G, H), while anti-CXCR2 increased both IFNγ^+^ CD8^+^ T cells and TNFα^+^ CD8^+^ T cells (Fig. 4I-L).

**Fig. 5:**
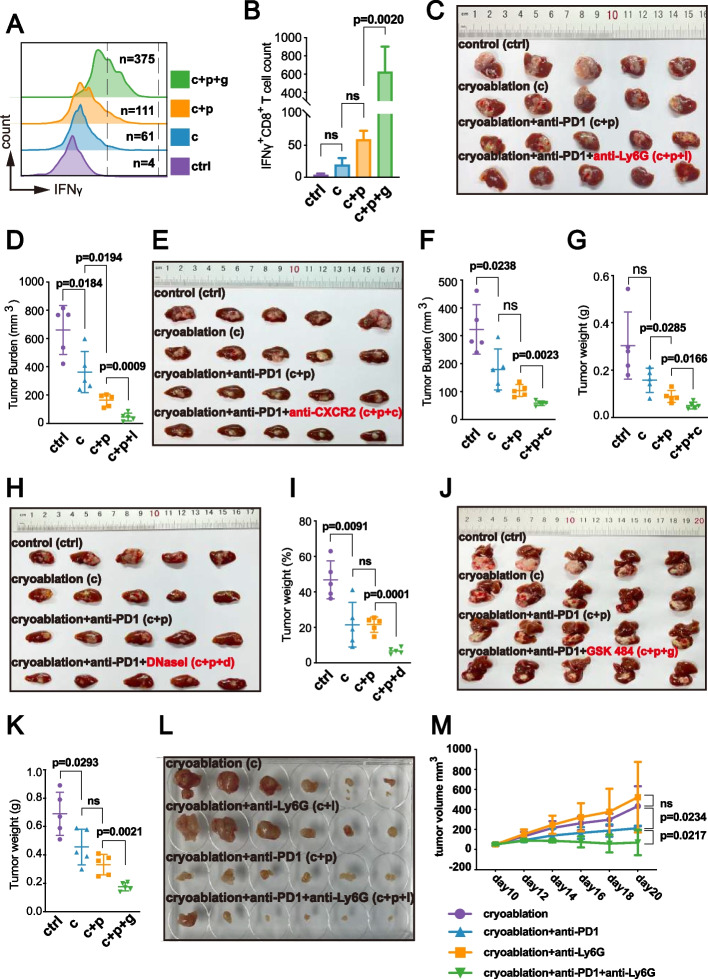
Neutrophil/NETs Inhibition Combined with Cryoablation and PD-1 Therapy Reduces Tumor Burden and Induces Abscopal Effects. **A**,** B** Flow cytometry plots and statistical analysis of IFNγ-expressing CD8^+^ T cell infiltration levels in the TME of the ctrl, c, c + p, and c + p + g groups (*n* = 5 per group, mean ± s.d., significance indicated by *p*-values, “ns” = no significant difference). **C** Photographs of tumors from the ctrl, c, c + p, and c + p + l groups. **D** Statistical analysis of tumor volume (mm^3^) in the ctrl, c, c + p, and c + p + l groups. Tumor volume was calculated using the ellipsoid formula (4/3 × π × length/2 × width/2 × width/2) after separating tumors from surrounding liver tissue. For samples with multiple tumors on a liver lobe, volumes were calculated separately and then summed to represent the total tumor burden for that sample. **E** Photographs of tumors from the ctrl, c, c + p, and c + p + c groups. **F**,** G** Statistical analysis of tumor volume (mm^3^) and weight (g) in the ctrl, c, c + p, and c + p + c groups. Tumor weight was measured post-separation from liver tissue. **H** Photographs of tumors from the ctrl, c, c + p, and c + p + d groups. **I** Statistical analysis of the tumor-to-liver lobe weight ratio (%) in the ctrl, c, c + p, and c + p + d groups. **J** Photographs of tumors from the ctrl, c, c + p, c + p + g groups. **K** Statistical analysis of tumor weight (g) in the ctrl, c, c + p, and c + p + g groups. **L**,** M** Exploration of the enhancement of abscopal effects through neutrophil depletion post-cryoablation. Experimental details are in the Supplementary Figure S6 and “Materials and Methods” section. **L** Photographs of contralateral tumors post-sacrifice. **M** Tumor growth curves during treatment. (*n* = 6 per group, mean ± s.d., significance indicated by *p*-values, “ns” = no significant difference). Neutrophil depletion enhanced the efficacy of post-cryoablation PD-1 antibody treatment, inducing abscopal effects

Administering the anti-PAD4 agent increased both CD8^+^ T cell infiltration and IFNγ expression following cryoablation combined with PD-1 antibody therapy (Figs. 4M, N, and 5A, B). Interestingly, the infiltration levels and cytotoxic effects of NK cells were not improved during this process (Supplementary Figure S5A-D), indicating that anti-PAD4 combined with PD-1 antibody mainly reshaped the adaptive immune response of CD8^+^ T cells post-cryoablation.

Our findings demonstrated that reducing neutrophil or NETs infiltration reshaped the TME and significantly delayed tumor progression (Fig. 5C-K). This discovery provided potential therapeutic targets for patients insensitive to cryoablation and PD-1 antibody therapy.

Previous studies have shown that enhancing sensitivity to PD-1 antibody triggers abscopal effects and improves prognosis in advanced colorectal cancer patients [32]. We designed a bilateral subcutaneous tumor-bearing mouse cohort study to determine if depleting neutrophils would trigger the abscopal effects of cryoablation combined with PD-1 antibody. Following complete cryoablation of one tumor in all mice, we monitored the growth of the untreated contralateral tumor to evaluate whether neutrophil depletion could induce abscopal effects.

Our observations revealed that anti-Ly6G administration markedly suppressed NETs formation within non-cryoablation lesions, significantly reducing tumor volume in the cryoablation + anti-PD1 + anti-Ly6G group compared to the cryoablation + anti-PD1 group (Fig. 5 L, M, Supplementary Figure S5E, F, Supplementary Figure S7C, D). This suggests that neutrophil depletion and inhibition of NETs formation may enhance abscopal effects.

### CXCL2-CXCR2-Ca^2+^-PAD4 Axis Regulates Neutrophil Recruitment and NETs Formation After Cryoablation

Cryoablation is known for its significant pro-inflammatory effects, but research on the regulatory mechanisms of neutrophil recruitment and NETs formation post-cryoablation is limited. To address this, we performed multi-omics analysis on mice with colorectal cancer liver metastases post-cryoablation.

**Fig. 6:**
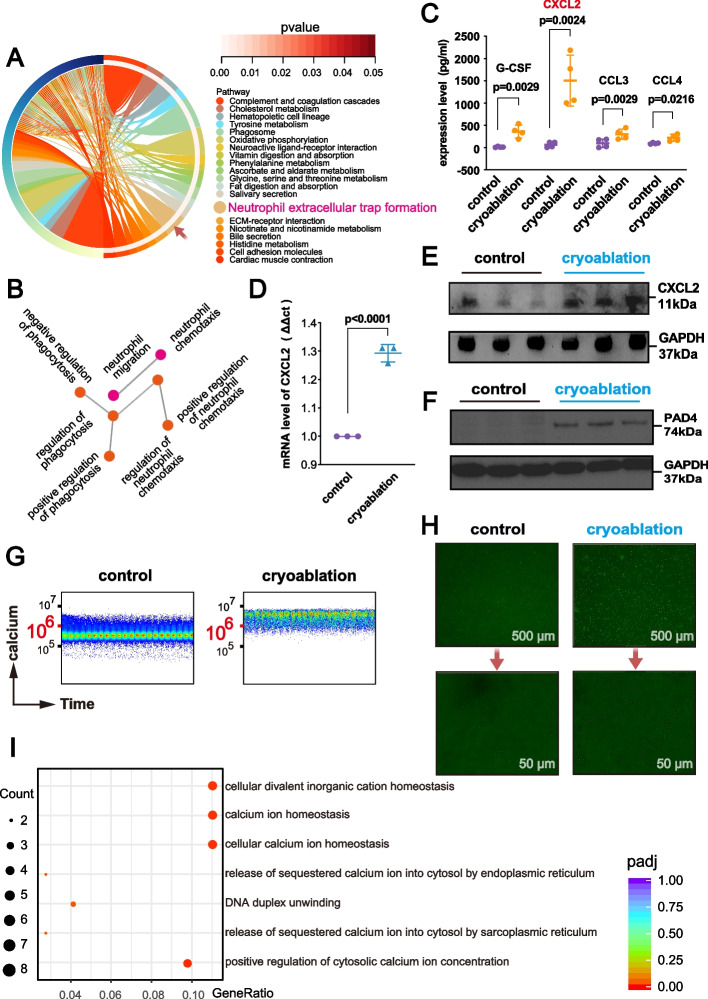
CXCL2-CXCR2-Ca^2+^-PAD4 Axis Drives Neutrophil Recruitment and NETs Formation Post-Cryoablation. **A** KEGG pathway enrichment analysis of 541 upregulated differentially expressed genes (DEGs) identified by 4D-Label-Free proteomics in liver metastasis tissues from mice 1 day post-cryoablation (*n* = 4) compared to control (*n* = 4), showing activation of NETs formation pathway. **B** GO enrichment analysis of the same DEGs shows significant enrichment in neutrophil chemotaxis and migration pathways. **C** Quantitative analysis of G-CSF, CXCL2, CCL3, and CCL4 levels in the TME of colorectal cancer liver metastasis tissues post-cryoablation shows a significant increase in CXCL2 expression. Details on the 32 cytokines analyzed are in Supplementary Figure S1. **D**,** E** qPCR and western blot results from MC38 cells subjected to in vitro cryoablation (4 min at low temperature followed by 3 min at 50 °C, cultured for 72 h) show that colorectal cancer cells are a significant source of CXCL2 in the TME post-cryoablation (*n* = 3 replicates per group). **F** Western blot results from human neutrophils (5.0E + 06 cells) subjected to in vitro cryoablation (3 min at low temperature, 3 min at 45 °C, cultured for 48 h) show increased PAD4 protein expression post-cryoablation (*n* = 3 replicates per group). **G**,** H** Flow cytometry and fluorescence microscopy show a significant increase in intracellular Ca^2+^ concentration in human neutrophils post-cryoablation. Neutrophils were isolated and subjected to in vitro cryoablation simulation (3 min at low temperature followed by 3 min at 45 °C), filtered to remove dead cell clumps, and immediately incubated with the Ca^2+^ fluorescent probe (Fluo 4-AM). After incubation and washing, the cells were observed under a fluorescence microscope and analyzed by flow cytometry using the FITC channel. **I** Single-cell RNA sequencing analysis shows that DEGs in neutrophils post-cryoablation are enriched in pathways related to intracellular Ca^2+^ regulation and DNA instability

Proteomic analysis revealed enhanced neutrophil chemotactic capabilities and activation of the NETs formation pathway (Fig. 6A, B). Analysis of selected cytokines showed a notable increase in CXCL2 within the TME following cryoablation (Fig. 6C). qPCR (Fig. 6D) and western blotting (Fig. 6E) identified tumor cells as a significant source of CXCL2 post-cryoablation, suggesting that cryoablation induces tumor cells to secrete CXCL2, which interacts with CXCR2 on neutrophils, increasing their infiltration.

Previous studies have shown that nuclear translocation of PAD4 and citrullination of histone H3 arginine residues are key mechanism for NETs formation [33–36]. Under physiological conditions, low intracellular Ca^2+^ concentration due to Ca^2+^ homeostasis limits PAD4 nuclear translocation [37]. We observed a significant increase in PAD4 protein expression in human neutrophils following cryoablation (Fig. 6F), along with a substantial rise in intracellular Ca^2+^ concentration (Fig. 6G-I). These findings suggest that increased intracellular Ca^2+^ concentration in neutrophils after cryoablation mediates PAD4 nuclear translocation, a key factor in NETs formation.

## Discussion

Our previous research showed that combining cryoablation with PD-1 antibody for liver metastases achieved higher efficacy (26.7%) compared to PD-1 monotherapy and holds promise for systemic control of distant metastases [11]. However, many patients with liver metastases experience resistance, highlighting the need to investigate the mechanisms underlying the heterogeneity of immune responses. We analyzed the dynamics of inflammatory cells during necrotic inflammation post-cryoablation. Neutrophils massively infiltrated ablated lesions as early as day 1 post-cryoablation and persisted for at least 14 days, suggesting a non-transient impact on the TME. Systemic increases in neutrophils in patients’ peripheral blood post-cryoablation indicated a systemic inflammatory response or a shift in hematopoietic cells towards the myeloid lineage.

The role of neutrophils and NETs has attracted significant interest [38, 39]. N1-type neutrophils participate in controlling tumor growth through various mechanisms, including cytotoxicity against tumor cells, tumor rejection, induction of tumor cell apoptosis, inhibition of metastasis, and enhancement of anti-tumor immune responses. In contrast, N2-type neutrophils are known for their significant roles in promoting tumor cell proliferation, oxidative damage, angiogenesis, NETs formation, and T cell suppression. Their contribution to tumorigenesis and tumor growth has been well established [25]. Our findings revealed no significant change in N1-type neutrophils within the TME pre- and post-cryoablation, while N2-type neutrophils markedly increased post-cryoablation, underscoring their pivotal role in colorectal cancer liver metastases. Characterizing tumor-associated neutrophil subsets in cryoablated tumors, understanding their immunophenotype and functions, and deciphering the molecular pathways of NETs formation post-cryoablation are essential. These insights are instrumental in refining synergistic therapeutic modalities.

To our knowledge, this is the first study reporting time-series data on neutrophils post-cryoablation in cancer, exploring the dynamic evolution of the TME. Our research uncovered two significant features of neutrophils post-cryoablation in colorectal cancer liver metastasis: temporal continuity and the chemotactic nature of immunosuppressive N2-type neutrophils, suggesting a sustained inhibitory effect on CD8^+^ T cells for 14 days or longer post-cryoablation.

Cryoablation increased intracellular Ca^2+^ levels in neutrophils, leading to NETs formation and subsequent impairment of anti-tumor immunity. Inhibiting neutrophils or NETs enhances the expression of cytotoxic proteins like IFNγ and TNFα in CD8^+^ T cells, resulting in better tumor control. These studies establish neutrophils and their NETs as potential targets for improving the efficacy of PD-1 antibody therapy post-cryoablation in colorectal cancer liver metastasis. Analysis of these datasets revealed the causal relationship between cryoablation, neutrophils, NETs, and T cells.

The combination of cryoablation with anti-PD1 treatment effectively slows tumor progression, indicating that administering anti-PD1 following cryoablation remains essential. Furthermore, the treatment strategy of cryoablation combined with anti-PD1 and anti-Ly6G further delays tumor progression and stabilizes tumor volume, suggesting that NETs may serve as a potential target for enhancing immunotherapy efficacy post-cryoablation.

Our study results demonstrate that the combination of cryoablation and anti-Ly6G requires the presence of a PD1 antibody to achieve optimal antitumor immune effects. From our findings, it is evident that cryoablation combined with anti-PD1 shows significantly better efficacy than cryoablation alone. Furthermore, in the presence of anti-Ly6G, the combination of cryoablation and PD1 antibody can further enhance the immunotherapeutic efficacy, suggesting that the increase in NETs induced by cryoablation is a critical factor influencing the effectiveness of cryoablation combined with PD1 antibody. However, in the absence of a PD1 antibody, although anti-Ly6G significantly inhibits NETs formation, it is insufficient to produce significant therapeutic effects.

In fact, numerous studies have confirmed that the physical barrier formed by NETs is a major reason for the low response rates in tumor immunotherapy. Tumor-killing cells (such as CD8 + T cells or NK cells) are hindered by the net-like structure of NETs, making it difficult for them to directly contact tumor cells, thereby impairing their tumor-killing function [40–42]. However, even after the NETs barrier is eliminated, and tumor cells become more accessible to immune cells, the increased binding between the PD-L1 on tumor cells and PD-1 on immune cells may suppress the tumor-killing function of immune cells. Therefore, in our study, the combination of cryoablation and anti-Ly6G does not exhibit ideal therapeutic efficacy, which likely requires the intervention of a PD1 antibody to achieve optimal effects.

## Conclusion

The treatment of colorectal cancer with liver metastases remains a significant challenge. Our study highlights that comprehensively characterizing the features of N2-type neutrophils and their inflammatory responses following cryoablation is a crucial strategy to improve the efficacy of immunotherapy for colorectal cancer with liver metastases. Furthermore, our data indicate that neutrophils not only reshape the immune microenvironment as individual entities but also that the impact of NETs derived from these cells on the immune microenvironment cannot be overlooked. These findings suggest the need to reassess the characteristics of neutrophil-mediated inflammatory responses following cryoablation, with particular emphasis on the critical role of the CXCL2-CXCR2-Ca^2+^-PAD4 axis in influencing the therapeutic efficacy of PD-1 antibodies in this context. This discovery offers promising potential for improving the treatment outcomes of colorectal cancer with liver metastases.

## Supplementary Information

Supplementary Material 1: Figure S1. Quantitative detection results of 32 cytokines in the TME of colorectal cancer liver metastasis in mice 24 h post-cryoablation. The experiment included two groups: cryoablation (*n* = 4) and control (*n* = 4). Data are shown as mean ± s.d., with each dot representing an independent sample. Significance of differences is indicated by *p*-values, with cytokines showing statistically significant differences (*p* < 0.05) marked in red. The expression levels of the 32 cytokines were detected using Luminex xMAP® liquid suspension array technology. Briefly, polystyrene microspheres with a diameter of 5.6 µm were dyed into different fluorescent colors by two red classification fluorescent dyes in different proportions to obtain 100 types of fluorescently coded microspheres. Antibodies or gene probes for different targets were covalently cross-linked to specific coded microspheres. The fluorescently coded microspheres were mixed with the target substances to form complexes and then reacted with labeled fluorophores. The microspheres, driven by sheath fluid, passed sequentially through red and green lasers (the red laser determined the fluorescence code of the microsphere, and the green laser measured the fluorescence intensity). The instrument read the fluorescence values, and the cytokine concentrations in the samples were calculated using a fitting curve.

Supplementary Material 2: Figure S2. (A) Diagram illustrating the in vitro simulation of cryoablation. (B) Flow cytometry images of IFNγ, perforin, TNFα, and PD-1 in CD8 + T cells (Viable CD45 + CD4 − CD8 + ) within the TME of mice with colorectal cancer liver metastasis at days 1, 5, and 14 post-cryoablation compared to the sham-operated control group. (C) Statistical analysis of the number of CD8 + T cells expressing TNFα. Each group consists of 5 independent biological samples. Data are expressed as mean ± s.d., with significance indicated by *p*-values, and “ns” signifies no significant difference.

Supplementary Material 3: Figure S3. (A, B) Representative flow cytometry images and statistical results demonstrate the enhanced proliferation capacity of CD8 + T cells (CFSE) after cryoablation, indicating that cryoablation-treated SW480 colorectal cancer cells can stimulate CD8 + T cell proliferation in vitro. In this experiment, SW480 cells or cryoablation-treated SW480 cells were co-cultured with human PBMCs in 12-well plates for 7 days. The groups were as follows: control (PBMC only), SW480 + PBMC, and SW480 (cryoablation) + PBMC. Human PBMCs were isolated using Lymphoprep™ (StemCell) and labeled with CFSE dye (5 μM, Abcam), then co-cultured with SW480 or cryoablation-treated SW480 cells (rapid freezing for 4 min, rapid thawing at 50 °C for 3 min), with three replicates per group. During the culture, anti-CD3/CD28 (25 μl, StemCell) and IL-2 (10 ng/ml, Peprotech) were used to simulate CD8 + T cells. After 7 days, cells were collected, stained with a viability dye and anti-human CD8 antibody (PE-Cy7, BD), and analyzed by flow cytometry. (C, D) Representative flow cytometry images and statistical results show that neutrophils suppress CD8 + T cells proliferation after cryoablation. The groups were SW480 (cryoablation) + PBMC and SW480 (cryoablation) + PBMC + neutrophils. Human PBMCs were isolated using Lymphoprep™ (StemCell) and labeled with CFSE dye (5 μM, Abcam), then co-cultured with cryoablation-treated SW480 cells (rapid freezing for 4 min, rapid thawing at 50 °C for 3 min). For the SW480 (cryoablation) + PBMC + neutrophils group, an additional 1.0E + 06 neutrophils (purified using the Human Neutrophil Isolation Kit, StemCell) were added. Three replicates were set for each group. During the culture, anti-CD3/CD28 (25 μl, StemCell) and IL-2 (10 ng/ml, Peprotech) were used to simulate CD8 + T cells, and 1.0E + 06 neutrophils were added every other day. After 7 days, cells were collected, stained with a viability dye and anti-human CD8 antibody (PE-Cy7, BD), and analyzed by flow cytometry. (E, F) show that neutrophils enhance apoptosis in CD8 + T cells after cryoablation. The experiment consisted of two groups: SW480 (cryoablation) + PBMC and SW480 (cryoablation) + PBMC + neutrophils. Briefly, 300,000 cryoablation-treated SW480 cells (rapid freezing for 4 min, rapid thawing at 50 °C for 3 min) and 300,000 human PBMCs (with or without 500,000 human neutrophils) were added 6-well plates. Anti-CD3/CD28 (25 μl) and IL-2 (10 ng/ml) were included in the co-culture system. After 5 days of culture, cells were collected and stained with anti-Annexin V and PI dyes according to the manufacturer’s instructions (GOONIE, Cat# 100–102). Each group included 3 replicates, and significant differences were indicated by *p*-values. Human PBMCs (Cat# 07851) and neutrophils (Cat# 19666) were purified from human peripheral blood following the manufacturer’s instructions (StemCell).

Supplementary Material 4: Figure S4. (A, B) Co-culture results show that cryoablation enhances the cytotoxic effects of CD8 + T cells, whereas (C, D) show that neutrophils significantly suppress the cytotoxic effects of CD8 + T cells post-cryoablation. The experimental design, cryoablation parameters, and cell quantities for (A) and (B) were consistent with Supplementary Figure S3 (A) and (B), and (C) and (D) were consistent with Supplementary Figure S3 (C) and (D). Before flow cytometry, all samples were treated with the Leukocyte Activation Cocktail (2 μl/ml, BD) for 4 h, followed by incubation with flow cytometry antibodies. Specifically, after staining with a viability dye, cells were incubated with anti-human CD8 (FITC, BioLegend) and CD4 (BV650, BD), then fixed and permeabilized, and further incubated with anti-human granzyme B (PE-Cy7, BioLegend), IFNγ (BV421, BD), and TNFα (PE, BD). Data are presented as mean ± s.d., with significance indicated by *p*-values, “ns” denotes no significant difference. (E) Dot plots illustrating the spatial relationships between neutrophils/NETs and CD8 + T cells in the TME of liver metastases in mice with colorectal cancer at days 1, 5, and 14 post-cryoablation, compared to the sham-operated control group, analyzed using Halo software. Briefly, pathological specimens were labeled with specific immunofluorescent dyes (CD11b and Ly6G double-positive cells as neutrophils; histone H3 and neutrophil elastase double-positive cells as NETs; CD8-positive cells as CD8 + T cells). Following immunofluorescent staining and scanning, the data were imported into Halo software. Cell identification parameters were set following the manufacturer’s guidelines, and the spatial analysis function projected neutrophils/NETs and CD8 + T cells as dots on a two-dimensional plane. The relative spatial distance between CD8 + T cells and neutrophils/NETs was calculated (unit: μm). In the figure, red dots represent CD8 + T cells within 50 μm of neutrophils/NETs, green dots represent CD8 + T cells more than 50 μm away, and blue dots represent neutrophils/NETs.

Supplementary Material 5: Figure S5. (A) Representative flow cytometry images illustrating differences in NK cells (defined as live CD45 + NK1.1 + ) infiltration within the TME of mice with colorectal cancer liver metastasis post-cryoablation in the ctrl, c, c + p, and c + p + g groups. (B) Flow cytometry plots showing differences in IFNγ expression in NK cells within the TME for the same groups. (C, D) present the statistical results of (A, B), respectively. Each group consists of 5 independent biological samples. Data are expressed as mean ± s.d., with *p*-values indicating significance, and “ns” denoting no significant difference. (E) Representative immunofluorescence images of NETs in the distant tumor (non-cryoablation) TME of mice with colorectal cancer liver metastasis in the c, c + l, c + p, and c + p + l groups. (F) presents the statistical analysis of (E). NETs are defined by the co-localization of neutrophils elastase (green, NE) and histone H3 (yellow) with DAPI staining for nuclei. Scale bar: 50 μm. Each group consists of 6 independent samples, with 3 regions of interest (ROIs) selected per sample (18 ROIs per group). Statistical analysis of NETs infiltration in the ROIs was performed using ImageJ software. Data are expressed as men ± s.d., with *p*-values indicating significance and “ns” denoting no significant difference. “c” represents the cryoablation group, “c + l” represents the cryoablation + anti-Ly6G group, “c + p” represents the cryoablation + anti-PD1 group, “c + p + l” represents the cryoablation + anti-PD1 + anti-Ly6G group.

Supplementary Material 6: Figure S6. This flowchart illustrates the experimental procedures and details for the five mouse cohorts designed in this study, displayed on a timeline. Purple dots represent cohort 1, blue dots represent cohort 2, yellow dots represent cohort 3, green dots represent cohort 4, and red dots represent cohort 5. The specific groupings for each cohort are indicated in the figure. In cohorts 1 to 4, each group consists of 5 independent samples, whereas in cohort 5, each group consists of 6 independent samples. Additionally, the liver metastasis model for colorectal cancer in mice for cohorts 1 to 4 was established by subcapsular injection of mouse colorectal cancer cells (MC38), utilizing a partial ablation strategy for cryoablation. In cohort 5, the mice were modeled by bilateral subcutaneous injection of MC38 cells, with complete cryoablation of the left tumor, while the right tumor did not receive cryoablation. For drug administration, in groups requiring combination therapy, such as the cryoablation + anti-PD1 + anti-Ly6G group in cohort 1, both drugs were administered simultaneously on days 13, 14, 15, 17, and 19. This principle applies to combination therapy groups in the other cohorts as well. Furthermore, the administration of the same drug (e.g., anti-PD1) across different cohorts was maintained at the same dosage. Specific details on drug dosages, administration routes, complete and partial cryoablation parameters, etc., are provided in the “Materials and Methods” section.

Supplementary Material 7: Figure S7. (A) Immunofluorescence images of NE (green) and H2B (red) in colorectal cancer liver metastasis tumor tissues of mice at 1, 5, and 14 days post-cryoablation, as well as in the control group. Hoechst 33342 (blue) indicates nuclei. Cells with colocalization of NE and H2B represent NETs. Scale bar: 50 μm. A total of 19 samples were analyzed, including samples from 1 day post-cryoablation (*n* = 5), 5 days post-cryoablation (*n* = 5), 14 days post-cryoablation (*n* = 4), and the control group (*n* = 5). Statistical analysis of the immunofluorescence staining results was performed using ImageJ software, and the results are shown in (B). (C) Representative immunofluorescence images of NETs in the tumor microenvironment (TME) of contralateral (non-cryoablated) tumors from colorectal cancer liver metastasis mice in the c, c + p, c + l, and c + p + l groups. Hoechst 33342 (blue) indicates nuclei, and the colocalization of NE (green) and H2B (red) indicates NETs. Scale bar: 50 μm. Each group included 6 independent samples, with one region of interest (ROI) selected per sample (6 ROIs per group). Statistical analysis of NETs infiltration in the distal non-cryoablated lesions was performed using ImageJ software, and the results are shown in (D). Data are expressed as men ± s.d., with *p*-values indicating significance and “ns” denoting no significant difference. “c” represents the cryoablation group, “c + l” represents the cryoablation + anti-Ly6G group, “c + p” represents the cryoablation + anti-PD1 group, “c + p + l” represents the cryoablation + anti-PD1 + anti-Ly6G group.

Supplementary Material 8: Table S1. This table presents the hub gene sets used for the GSVA scores of various neutrophil subpopulations in this study. The gene sets were selected based on the reference [43]. Briefly, the R package “GSVA” was used to conduct the GSVA analysis. An enrichment score was calculated for each cluster using a non-parametric, unsupervised approach that transforms a traditional gene matrix. The mean values of the enrichment scores of cells were then compared using the t-test. A false discovery rate of < 0.25 was considered significant.

Supplementary Material 9: Table S2. This table provides detailed information on the usage of multi-omics data obtained in this study. “tumor 1” and “tumor 2” refer to the two tumors inoculated on the liver lobes during the construction of colorectal cancer liver metastases. Tumor 1 denotes the tumor that underwent cryoablation, whereas tumor 2 denotes the tumor that did not undergo cryoablation.

## Data Availability

The datasets utilized and/or analyzed in this study can be obtained from the corresponding authors upon reasonable request.

## Abbreviations

Ca^2+^: Calcium Ion
NETs: Neutrophil Extracellular Traps
Fas: Factor-Related Apoptosis
TNF-α: Tumor Necrosis Factor
ICAM-1: Intercellular Cell Adhesion Molecule-1
NK cell: Natural Killer Cell
Arg-1: Arginase-1
Arg-2: Arginase-2
VEGF: Vascular Endothelial Growth Factor
MMP-9: Matrix Metalloproteinase-9
TLR: Toll-Like Receptor
PD-1: Programmed Cell Death Protein 1
PD-L1: Programmed Death-Ligand 1
CTLA-4: Cytotoxic T-Lymphocyte Antigen 4
TME: Tumor Microenvironment
NLR: Neutrophil to Lymphocyte Ratio
GSVA: Gene Set Variation Analysis
G-CSF: Granulocyte Colony-Stimulating Factor
GM-CSF: Granulocyte–Macrophage Colony-Stimulating Factor
M-CSF: Macrophage Colony-Stimulating Factor
CXCL1: C-X-C Motif Chemokine Ligand 1
CXCL2: C-X-C Motif Chemokine Ligand 2
CXCL5: C-X-C Motif Chemokine Ligand 5
CXCL9: C-X-C Motif Chemokine Ligand 9
CXCL10: C-X-C Motif Chemokine Ligand 10
CCL2: C–C Motif Chemokine Ligand 2
CCL3: C–C Motif Chemokine Ligand 3
CCL4: C–C Motif Chemokine Ligand 4
CCL5: C–C Motif Chemokine Ligand 5
CCL11: C–C Motif Chemokine Ligand 11
IL-1α: Interleukin-1 Alpha
IL-1β: Interleukin-1 Beta
IL-2: Interleukin-2
IL-3: Interleukin-3
IL-4: Interleukin-4
IL-5: Interleukin-5
IL-6: Interleukin-6
IL-7: Interleukin-7
IL-9: Interleukin-9
IL-10: Interleukin-10
IL-12p40: Interleukin-12 Subunit p40
IL-12p70: Interleukin-12 Active Heterodimeric Cytokine
IL-13: Interleukin-13
IL-15: Interleukin-15
IL-17: Interleukin-17
TNF-α: Tumor Necrosis Factor Alpha
LIF: Leukemia Inhibitory Factor
IFN-γ: Interferon Gamma
ATCC: American Type Culture Collection
HL-60 cell: Human Promyelocytic Leukemia Cell
dHL60 cell: HL-60 cells differentiated into neutrophil-like cells
DMSO: Dimethyl Sulfoxide
PBS: Phosphate Buffer Saline
DNase I: Deoxyribonuclease I
PAD4: Peptidylarginine Deiminase 4
TGF-β: Transforming Growth Factor Beta
S100A8: S100 Calcium Binding Protein A8
S100A9: S100 Calcium Binding Protein A9
NE: Neutrophil Elastase
H3: Histone H3
H2B: Histone H2B
