# Supplementary figures and images for: Cryoablation-induced neutrophil Ca2+ elevation and NET formation exacerbate immune escape in colorectal cancer liver metastasis
Source: J Exp Clin Cancer Res. 2024 Dec 9;43:319. doi: 10.1186/s13046-024-03244-z (PMC11626751; doi:10.1186/s13046-024-03244-z)

# Supplementary Figure S1

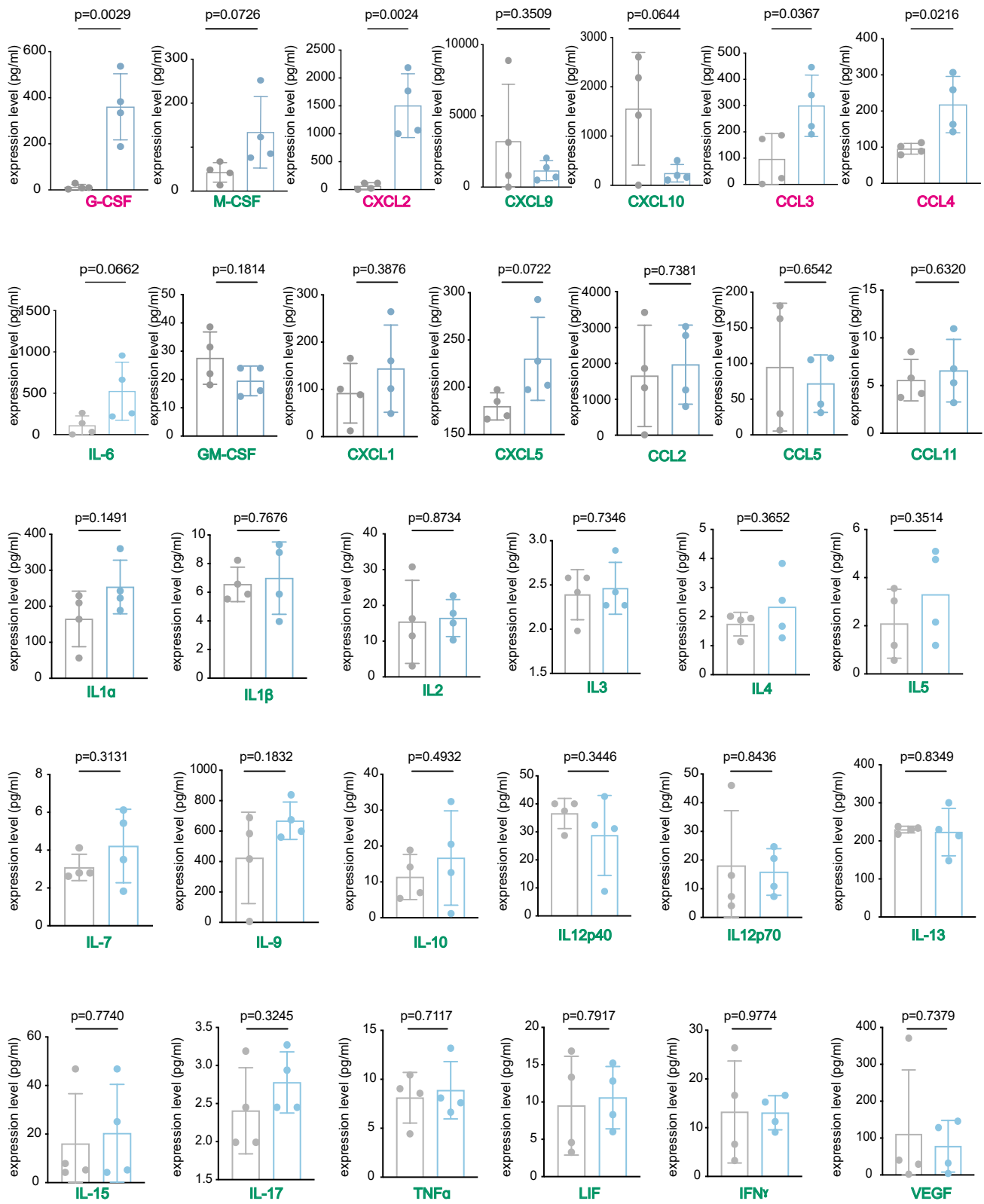

Supplement: Supplementary file 1 — Supplementary Material 1: Figure S1. Quantitative detection results of 32 cytokines in the TME of colorectal cancer liver metastasis in mice 24 h post-cryoablation. The experiment included two groups: cryoablation (n = 4) and control (n = 4). Data are shown as mean ± s.d., with each dot representing an independent sample. Significance of differences is indicated by p-values, with cytokines showing statistically significant differences (p < 0.05) marked in red. The expression levels of the 32 cytokines were detected using Luminex xMAP® liquid suspension array technology. Briefly, polystyrene microspheres with a diameter of 5.6 µm were dyed into different fluorescent colors by two red classification fluorescent dyes in different proportions to obtain 100 types of fluorescently coded microspheres. Antibodies or gene probes for different targets were covalently cross-linked to specific coded microspheres. The fluorescently coded microspheres were mixed with the target substances to form complexes and then reacted with labeled fluorophores. The microspheres, driven by sheath fluid, passed sequentially through red and green lasers (the red laser determined the fluorescence code of the microsphere, and the green laser measured the fluorescence intensity). The instrument read the fluorescence values, and the cytokine concentrations in the samples were calculated using a fitting curve. [file 13046_2024_3244_MOESM1_ESM.pdf]

# Supplementary Figure S2

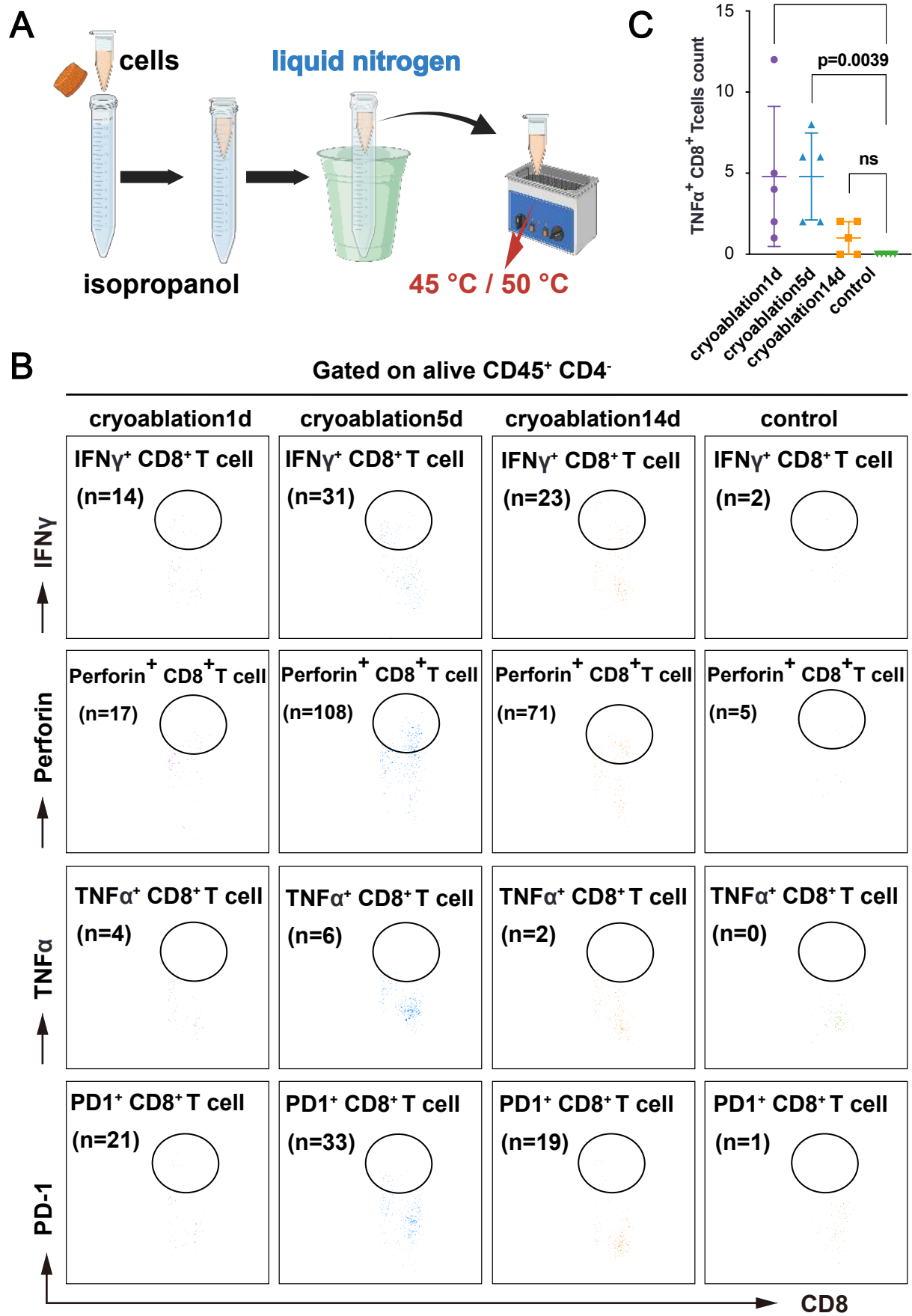

Supplement: Supplementary file 2 — Supplementary Material 2: Figure S2. (A) Diagram illustrating the in vitro simulation of cryoablation. (B) Flow cytometry images of IFNγ, perforin, TNFα, and PD-1 in CD8 + T cells (Viable CD45 + CD4 − CD8 + ) within the TME of mice with colorectal cancer liver metastasis at days 1, 5, and 14 post-cryoablation compared to the sham-operated control group. (C) Statistical analysis of the number of CD8 + T cells expressing TNFα. Each group consists of 5 independent biological samples. Data are expressed as mean ± s.d., with significance indicated by p-values, and “ns” signifies no significant difference. [file 13046_2024_3244_MOESM2_ESM.pdf]

Supplementary Figure S3

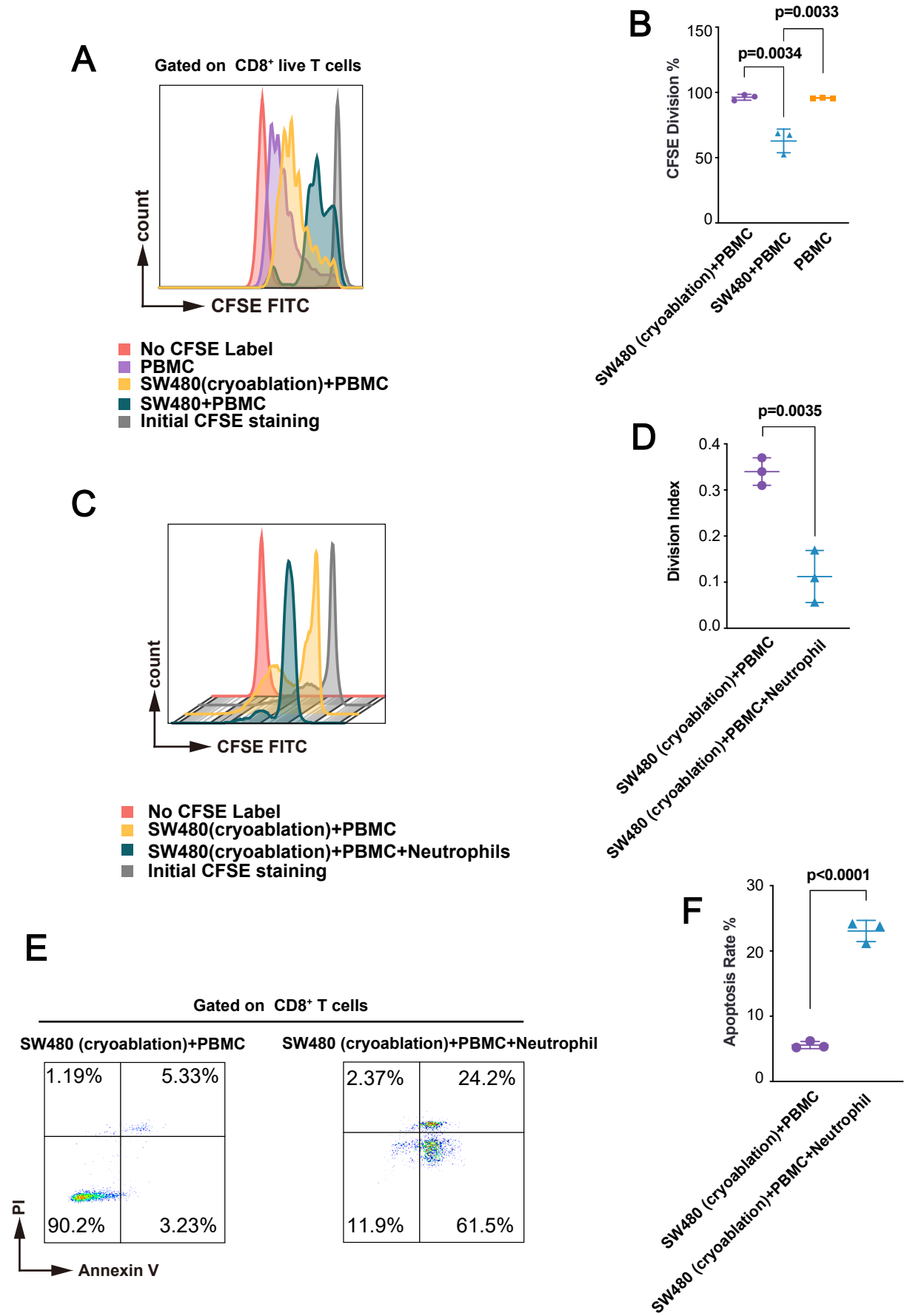

Supplement: Supplementary file 3 — Supplementary Material 3: Figure S3. (A, B) Representative flow cytometry images and statistical results demonstrate the enhanced proliferation capacity of CD8 + T cells (CFSE) after cryoablation, indicating that cryoablation-treated SW480 colorectal cancer cells can stimulate CD8 + T cell proliferation in vitro. In this experiment, SW480 cells or cryoablation-treated SW480 cells were co-cultured with human PBMCs in 12-well plates for 7 days. The groups were as follows: control (PBMC only), SW480 + PBMC, and SW480 (cryoablation) + PBMC. Human PBMCs were isolated using Lymphoprep™ (StemCell) and labeled with CFSE dye (5 μM, Abcam), then co-cultured with SW480 or cryoablation-treated SW480 cells (rapid freezing for 4 min, rapid thawing at 50 °C for 3 min), with three replicates per group. During the culture, anti-CD3/CD28 (25 μl, StemCell) and IL-2 (10 ng/ml, Peprotech) were used to simulate CD8 + T cells. After 7 days, cells were collected, stained with a viability dye and anti-human CD8 antibody (PE-Cy7, BD), and analyzed by flow cytometry. (C, D) Representative flow cytometry images and statistical results show that neutrophils suppress CD8 + T cells proliferation after cryoablation. The groups were SW480 (cryoablation) + PBMC and SW480 (cryoablation) + PBMC + neutrophils. Human PBMCs were isolated using Lymphoprep™ (StemCell) and labeled with CFSE dye (5 μM, Abcam), then co-cultured with cryoablation-treated SW480 cells (rapid freezing for 4 min, rapid thawing at 50 °C for 3 min). For the SW480 (cryoablation) + PBMC + neutrophils group, an additional 1.0E + 06 neutrophils (purified using the Human Neutrophil Isolation Kit, StemCell) were added. Three replicates were set for each group. During the culture, anti-CD3/CD28 (25 μl, StemCell) and IL-2 (10 ng/ml, Peprotech) were used to simulate CD8 + T cells, and 1.0E + 06 neutrophils were added every other day. After 7 days, cells were collected, stained with a viability dye and anti-human CD8 antibody (PE-Cy7, BD), an [file 13046_2024_3244_MOESM3_ESM.pdf]

Supplementary Figure S4

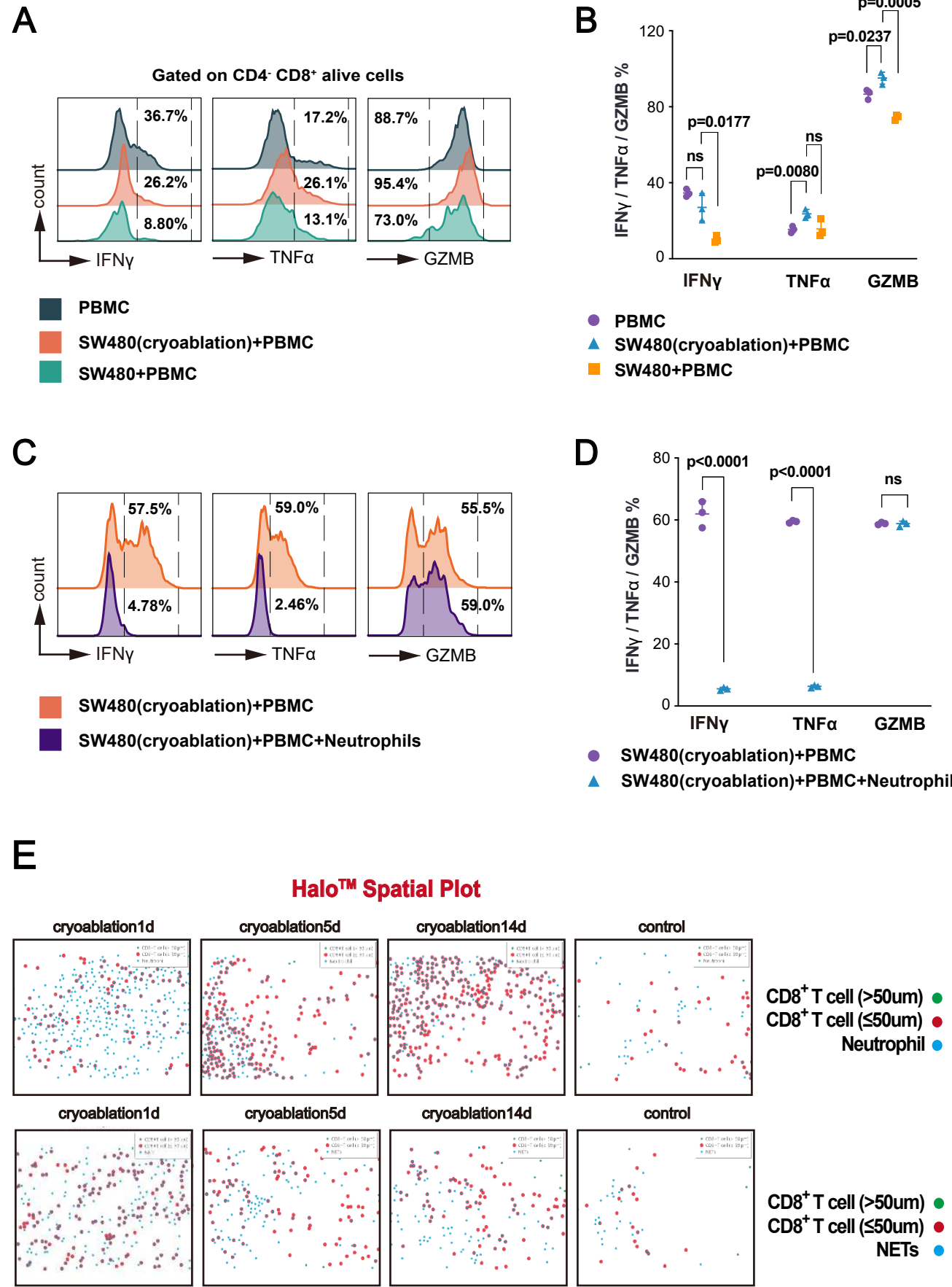

Supplement: Supplementary file 4 — Supplementary Material 4: Figure S4. (A, B) Co-culture results show that cryoablation enhances the cytotoxic effects of CD8 + T cells, whereas (C, D) show that neutrophils significantly suppress the cytotoxic effects of CD8 + T cells post-cryoablation. The experimental design, cryoablation parameters, and cell quantities for (A) and (B) were consistent with Supplementary Figure S3 (A) and (B), and (C) and (D) were consistent with Supplementary Figure S3 (C) and (D). Before flow cytometry, all samples were treated with the Leukocyte Activation Cocktail (2 μl/ml, BD) for 4 h, followed by incubation with flow cytometry antibodies. Specifically, after staining with a viability dye, cells were incubated with anti-human CD8 (FITC, BioLegend) and CD4 (BV650, BD), then fixed and permeabilized, and further incubated with anti-human granzyme B (PE-Cy7, BioLegend), IFNγ (BV421, BD), and TNFα (PE, BD). Data are presented as mean ± s.d., with significance indicated by p-values, “ns” denotes no significant difference. (E) Dot plots illustrating the spatial relationships between neutrophils/NETs and CD8 + T cells in the TME of liver metastases in mice with colorectal cancer at days 1, 5, and 14 post-cryoablation, compared to the sham-operated control group, analyzed using Halo software. Briefly, pathological specimens were labeled with specific immunofluorescent dyes (CD11b and Ly6G double-positive cells as neutrophils; histone H3 and neutrophil elastase double-positive cells as NETs; CD8-positive cells as CD8 + T cells). Following immunofluorescent staining and scanning, the data were imported into Halo software. Cell identification parameters were set following the manufacturer’s guidelines, and the spatial analysis function projected neutrophils/NETs and CD8 + T cells as dots on a two-dimensional plane. The relative spatial distance between CD8 + T cells and neutrophils/NETs was calculated (unit: μm). In the figure, red dots represent CD8 + T cells within 50 μm of neutrophils/N [file 13046_2024_3244_MOESM4_ESM.pdf]

Supplementary Figure S5

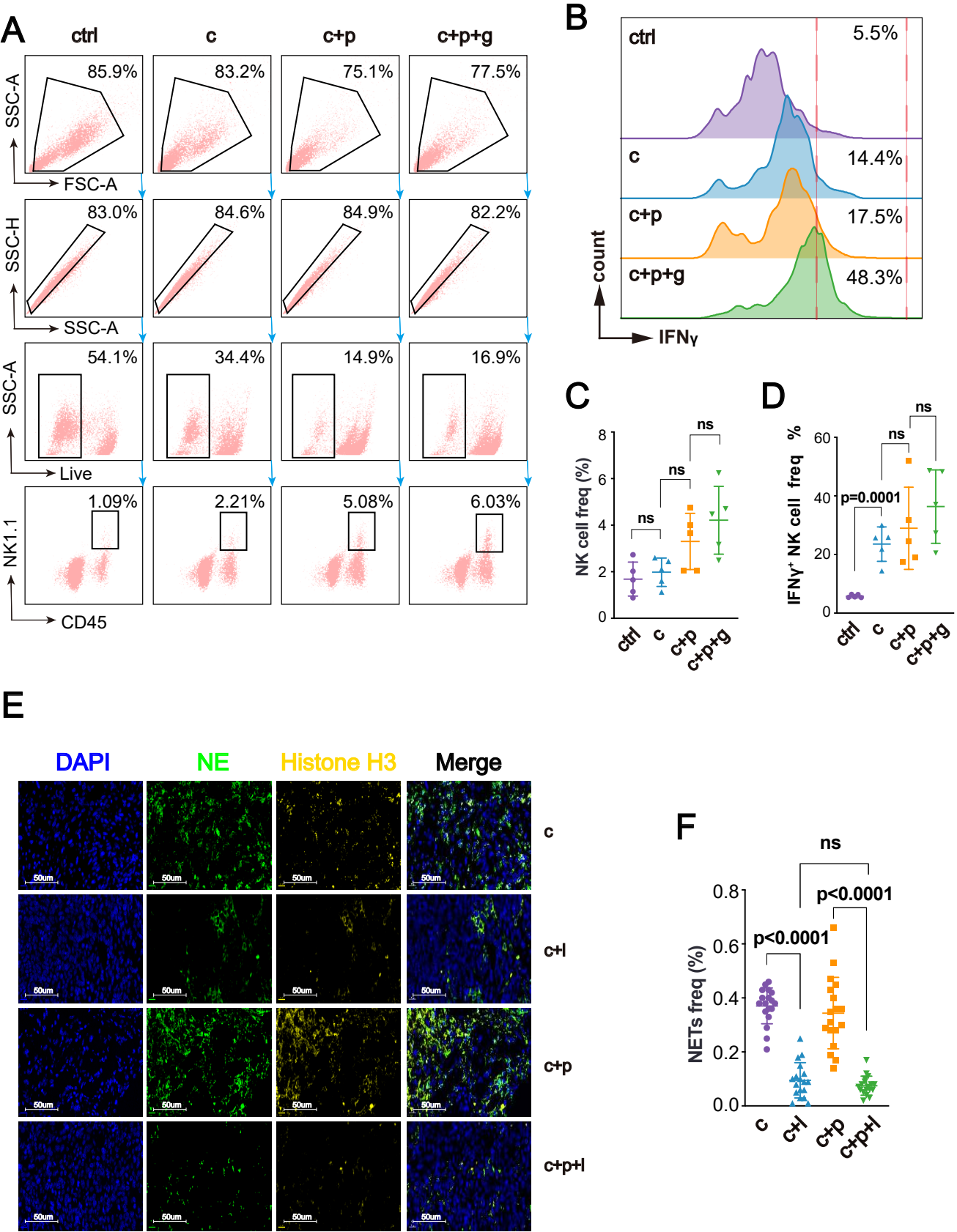

Supplement: Supplementary file 5 — Supplementary Material 5: Figure S5. (A) Representative flow cytometry images illustrating differences in NK cells (defined as live CD45 + NK1.1 + ) infiltration within the TME of mice with colorectal cancer liver metastasis post-cryoablation in the ctrl, c, c + p, and c + p + g groups. (B) Flow cytometry plots showing differences in IFNγ expression in NK cells within the TME for the same groups. (C, D) present the statistical results of (A, B), respectively. Each group consists of 5 independent biological samples. Data are expressed as mean ± s.d., with p-values indicating significance, and “ns” denoting no significant difference. (E) Representative immunofluorescence images of NETs in the distant tumor (non-cryoablation) TME of mice with colorectal cancer liver metastasis in the c, c + l, c + p, and c + p + l groups. (F) presents the statistical analysis of (E). NETs are defined by the co-localization of neutrophils elastase (green, NE) and histone H3 (yellow) with DAPI staining for nuclei. Scale bar: 50 μm. Each group consists of 6 independent samples, with 3 regions of interest (ROIs) selected per sample (18 ROIs per group). Statistical analysis of NETs infiltration in the ROIs was performed using ImageJ software. Data are expressed as men ± s.d., with p-values indicating significance and “ns” denoting no significant difference. “c” represents the cryoablation group, “c + l” represents the cryoablation + anti-Ly6G group, “c + p” represents the cryoablation + anti-PD1 group, “c + p + l” represents the cryoablation + anti-PD1 + anti-Ly6G group. [file 13046_2024_3244_MOESM5_ESM.pdf]

Supplementary Figure S7

A

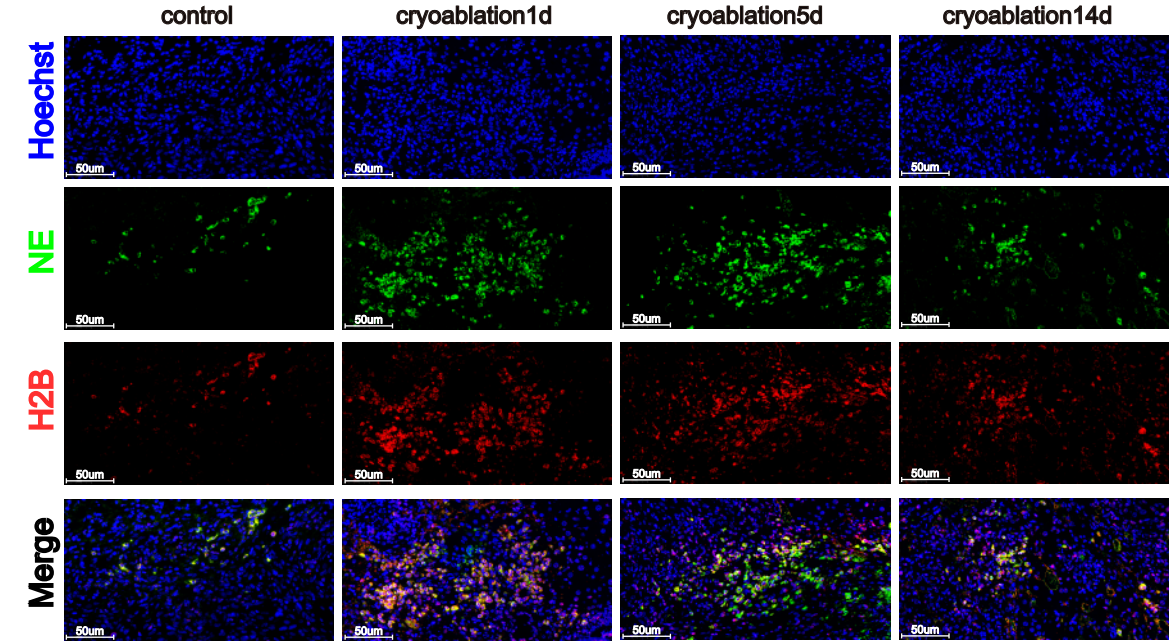

B

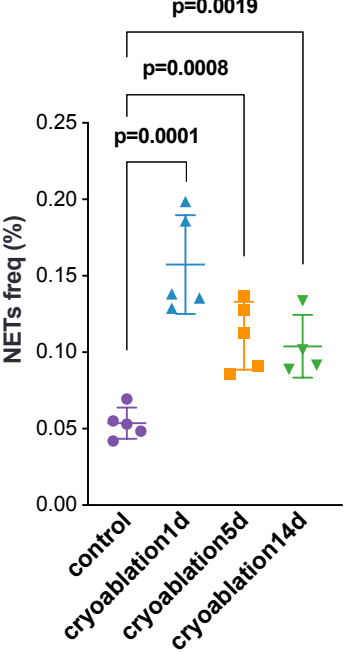

C

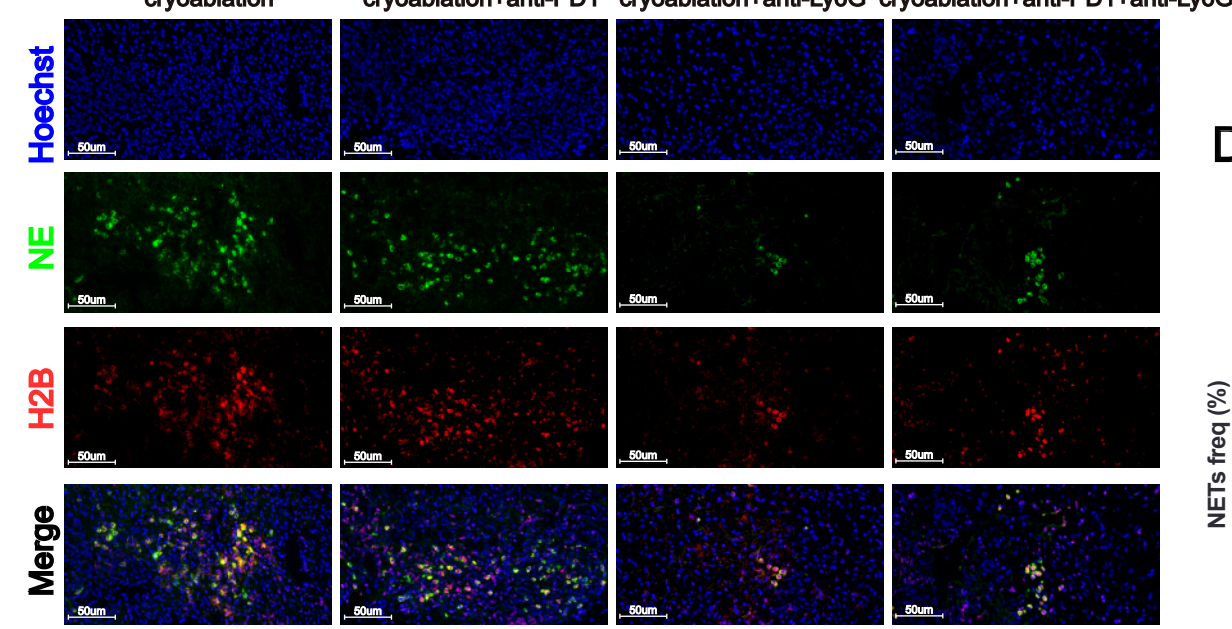

D

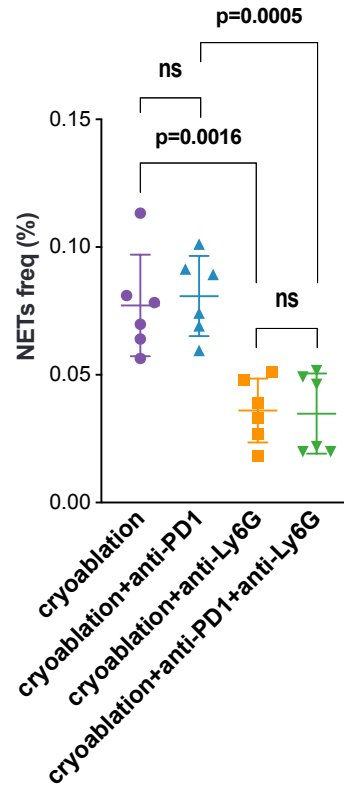

Supplement: Supplementary file 7 — Supplementary Material 7: Figure S7. (A) Immunofluorescence images of NE (green) and H2B (red) in colorectal cancer liver metastasis tumor tissues of mice at 1, 5, and 14 days post-cryoablation, as well as in the control group. Hoechst 33342 (blue) indicates nuclei. Cells with colocalization of NE and H2B represent NETs. Scale bar: 50 μm. A total of 19 samples were analyzed, including samples from 1 day post-cryoablation (n = 5), 5 days post-cryoablation (n = 5), 14 days post-cryoablation (n = 4), and the control group (n = 5). Statistical analysis of the immunofluorescence staining results was performed using ImageJ software, and the results are shown in (B). (C) Representative immunofluorescence images of NETs in the tumor microenvironment (TME) of contralateral (non-cryoablated) tumors from colorectal cancer liver metastasis mice in the c, c + p, c + l, and c + p + l groups. Hoechst 33342 (blue) indicates nuclei, and the colocalization of NE (green) and H2B (red) indicates NETs. Scale bar: 50 μm. Each group included 6 independent samples, with one region of interest (ROI) selected per sample (6 ROIs per group). Statistical analysis of NETs infiltration in the distal non-cryoablated lesions was performed using ImageJ software, and the results are shown in (D). Data are expressed as men ± s.d., with p-values indicating significance and “ns” denoting no significant difference. “c” represents the cryoablation group, “c + l” represents the cryoablation + anti-Ly6G group, “c + p” represents the cryoablation + anti-PD1 group, “c + p + l” represents the cryoablation + anti-PD1 + anti-Ly6G group. [file 13046_2024_3244_MOESM7_ESM.pdf]
