# Supplementary material for: Cryoablation-induced neutrophil Ca2+ elevation and NET formation exacerbate immune escape in colorectal cancer liver metastasis
Source: J Exp Clin Cancer Res. 2024 Dec 9;43:319. doi: 10.1186/s13046-024-03244-z (PMC11626751; doi:10.1186/s13046-024-03244-z)
Supplement: Supplementary file 6 — Supplementary Material 6: Figure S6. This flowchart illustrates the experimental procedures and details for the five mouse cohorts designed in this study, displayed on a timeline. Purple dots represent cohort 1, blue dots represent cohort 2, yellow dots represent cohort 3, green dots represent cohort 4, and red dots represent cohort 5. The specific groupings for each cohort are indicated in the figure. In cohorts 1 to 4, each group consists of 5 independent samples, whereas in cohort 5, each group consists of 6 independent samples. Additionally, the liver metastasis model for colorectal cancer in mice for cohorts 1 to 4 was established by subcapsular injection of mouse colorectal cancer cells (MC38), utilizing a partial ablation strategy for cryoablation. In cohort 5, the mice were modeled by bilateral subcutaneous injection of MC38 cells, with complete cryoablation of the left tumor, while the right tumor did not receive cryoablation. For drug administration, in groups requiring combination therapy, such as the cryoablation + anti-PD1 + anti-Ly6G group in cohort 1, both drugs were administered simultaneously on days 13, 14, 15, 17, and 19. This principle applies to combination therapy groups in the other cohorts as well. Furthermore, the administration of the same drug (e.g., anti-PD1) across different cohorts was maintained at the same dosage. Specific details on drug dosages, administration routes, complete and partial cryoablation parameters, etc., are provided in the “Materials and Methods” section. [file 13046_2024_3244_MOESM6_ESM.pdf]

Supplementary Figure S6

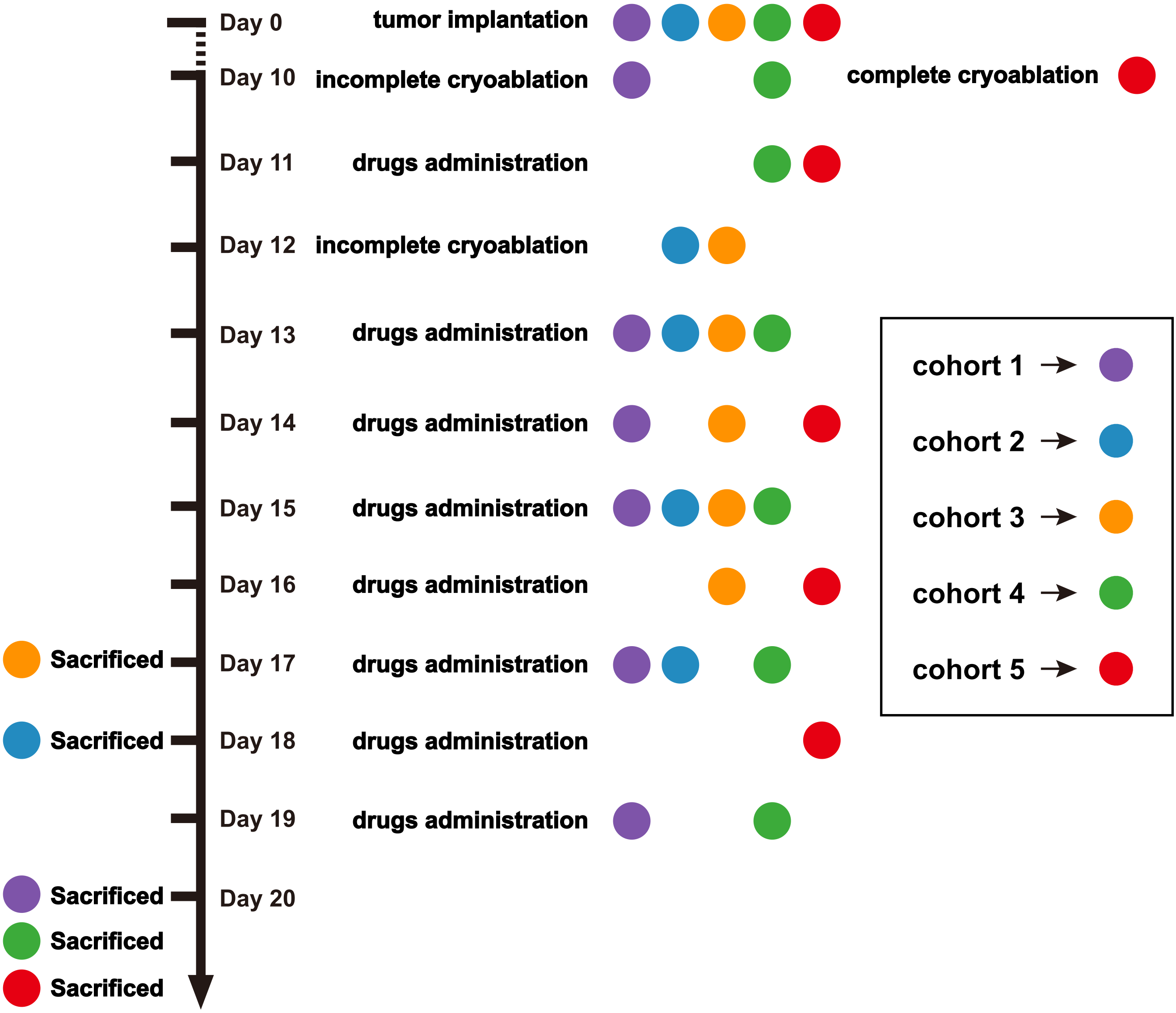

cohort 1 groups: control, cryoablation, cryoablation+anti-PD1, cryoablation+anti-PD1+anti-LyG

cohort 2 groups: control, cryoablation, cryoablation+anti-PD1, cryoablation+anti-PD1+anti-CXCR2

cohort 3 groups: control, cryoablation, cryoablation+anti-PD1, cryoablation+anti-PD1+DNase I

cohort 4 groups: control, cryoablation, cryoablation+anti-PD1, cryoablation+anti-PD1+GSK484 HCl

cohort 5 groups: cryoablation, cryoablation+anti-Ly6G, cryoablation+anti-PD1, cryoablation+anti-PD1+anti-ly6G
