# Supplementary material for: Cryoablation-induced neutrophil Ca2+ elevation and NET formation exacerbate immune escape in colorectal cancer liver metastasis
Source: J Exp Clin Cancer Res. 2024 Dec 9;43:319. doi: 10.1186/s13046-024-03244-z (PMC11626751; doi:10.1186/s13046-024-03244-z)
Supplement: Supplementary file 8 — Supplementary Material 8: Table S1. This table presents the hub gene sets used for the GSVA scores of various neutrophil subpopulations in this study. The gene sets were selected based on the reference [43]. Briefly, the R package “GSVA” was used to conduct the GSVA analysis. An enrichment score was calculated for each cluster using a non-parametric, unsupervised approach that transforms a traditional gene matrix. The mean values of the enrichment scores of cells were then compared using the t-test. A false discovery rate of < 0.25 was considered significant. [file 13046_2024_3244_MOESM8_ESM.pdf]

Supplementary Table S1

GSVA Gene Set

Gene ID			Description
HGNC symbol	Entrez Gene	Ensembl	(coding protein)
AKT1	207	ENSG00000142208	AKT serine/threonine kinase 1
AKT2	208	ENSG00000105221	AKT serine/threonine kinase 2
ATG7	10533	ENSG00000197548	Autophagy related 7
CLEC6A	93978	ENSG00000205846	Dectin-2
CSF3	1440	ENSG00000108342	Granulocyte colony stimulating factor
CTSG	1511	ENSG00000100448	Cathepsin G
CYBB	1536	ENSG00000165168	NADPH oxidase
DNASE1	1773	ENSG00000213938	Deoxyribonuclease I
ELANE	1991	ENSG00000197561	Neutrophil elastase
ENTPD4	14573	ENSG00000197217	Ectonucleoside Triphosphate Diphosphohydrolase 4
F3	2152	ENSG00000117525	Coagulation Factor III, tissue factor
HMGB1	3146	ENSG00000189403	High mobility group box 1
IL17A	3605	ENSG00000112115	Interleukin 17
IL1B	3553	ENSG00000125538	Interleukin 1 beta
IL6	3569	ENSG00000136244	Interleukin 6
IL8	3576	ENSG00000169429	Interleukin 8
IRAK4	51135	ENSG00000198001	Interleukin 1 receptor associated kinase 4
ITGAM	3684	ENSG00000169896	Complement component 3 receptor 3 subunit
ITGB2	3689	ENSG00000160255	Complement component 3 receptor 3 and 4 subunit
KCNN3	3782	ENSG00000143603	Potassium channel, calcium activated
MAPK1	5594	ENSG00000100030	Mitogen-activated protein kinase 1
MAPK3	5595	ENSG00000102882	Mitogen-activated protein kinase 3
MMP9	4218	ENSG00000100985	Matrix metallopeptidase 9
MPO	4353	ENSG00000005381	Myeloperoxidase
MTOR	2475	ENSG00000198793	Mechanistic target of rapamycin kinase
PADI4	23569	ENSG00000159339	Peptidyl arginine deiminase 4
PTAFR	5724	ENSG00000169403	Platelet activation factor receptor
PIK3CA	5290	ENSG00000121879	Phosphatidylinositol-4,5-bisphosphate 3-kinase
RIPK1	8737	ENSG00000137275	Receptor interacting serine/threonine kinase 1
RIPK3	11035	ENSG00000129465	Receptor interacting serine/threonine kinase 3
SELP	6403	ENSG00000174175	P-selectin
SELPLG	6404	ENSG00000110876	P-selectin receptor
SIGLEC14	10049587	ENSG00000254415	Sialic acid binding Ig like lectin 4
TLR2	7097	ENSG00000137462	Toll like receptor 2
TLR4	7099	ENSG00000136869	Toll like receptor 4
TLR7	51284	ENSG00000196664	Toll like receptor 7
TLR8	51311	ENSG00000101916	Toll like receptor 8
TNF	7124	ENSG00000232810	Tumor necrosis factor-alpha
