# Supplementary material for: Cryoablation-induced neutrophil Ca2+ elevation and NET formation exacerbate immune escape in colorectal cancer liver metastasis
Source: J Exp Clin Cancer Res. 2024 Dec 9;43:319. doi: 10.1186/s13046-024-03244-z (PMC11626751; doi:10.1186/s13046-024-03244-z)

Supplementary Table S2

MouseID	Group (SacrificedTime)	scRNA-seq	Bulk RNA-seq	Proteomic	32 Cytokines	IF Stain
#A12	1 day post-cryoablation	tumor 1	tumor 1	tumor 1	tumor 1	tumor 1
#A23		tumor 1	tumor 1			
#A09		tumor 1	tumor 1			tumor 1
#A27		tumor 1	tumor 1	tumor 1	tumor 1	tumor 1
#A22		tumor 1	tumor 1			
#A16		tumor 1	tumor 1			tumor 1
#A19		tumor 1	tumor 1			tumor 1
#A30		tumor 1	tumor 1	tumor 1	tumor 1	
#A31			tumor 1	tumor 1	tumor 1	
#A05			tumor 1			
#A06	5 days post-cryoablation	tumor 1	tumor 1			tumor 1
#A29		tumor 1	tumor 1			tumor 1
#A11		tumor 1	tumor 1			tumor 1
#A07		tumor 1	tumor 1			tumor 1
#A14		tumor 1	tumor 1			tumor 1
#A10			tumor 1			
#A34	14 days post-cryoablation	tumor 1+tumor 2	tumor 1+tumor 2			
#A33		tumor 1+tumor 2	tumor 1+tumor 2			
#A32		tumor 1+tumor 2	tumor 1+tumor 2			
#A21		tumor 1+tumor 2	tumor 1+tumor 2			tumor 1
#A18		tumor 1+tumor 2	tumor 1+tumor 2			tumor 1
#A08			tumor 1+tumor 2			tumor 1
#A17			tumor 1+tumor 2			tumor 1
#A15	sham-operation	tumor 1	tumor 1			tumor 1
#A24		tumor 1	tumor 1			tumor 1
#A13		tumor 1	tumor 1			tumor 1
#A20		tumor 1	tumor 1	tumor 1	tumor 1	tumor 1
#A25		tumor 1	tumor 1	tumor 1	tumor 1	
#A35		tumor 1	tumor 1	tumor 1	tumor 1	
#A28		tumor 1	tumor 1			tumor 1
#A26		tumor 1		tumor 1	tumor 1	
total:31mice						

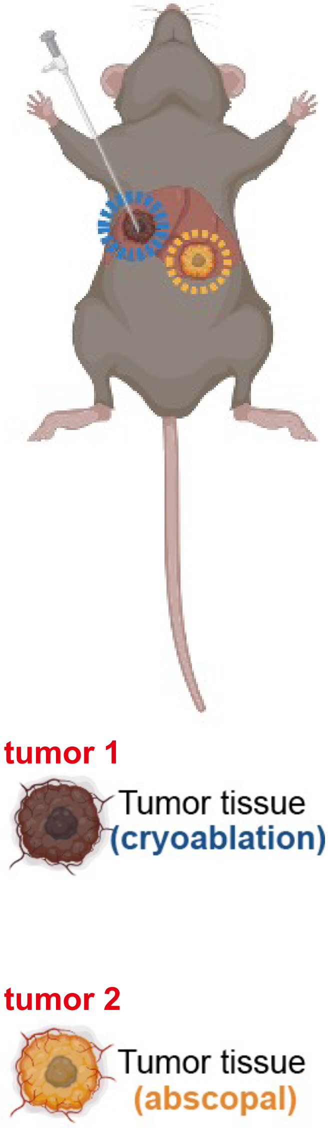

Supplement: Supplementary file 9 — Supplementary Material 9: Table S2. This table provides detailed information on the usage of multi-omics data obtained in this study. “tumor 1” and “tumor 2” refer to the two tumors inoculated on the liver lobes during the construction of colorectal cancer liver metastases. Tumor 1 denotes the tumor that underwent cryoablation, whereas tumor 2 denotes the tumor that did not undergo cryoablation. [file 13046_2024_3244_MOESM9_ESM.pdf]
